# Human and Bovine Milk Oligosaccharides Elicit Improved Recognition Memory Concurrent With Alterations in Regional Brain Volumes and Hippocampal mRNA Expression

**DOI:** 10.3389/fnins.2020.00770

**Published:** 2020-08-13

**Authors:** Stephen A. Fleming, Austin T. Mudd, Jonas Hauser, Jian Yan, Sylviane Metairon, Pascal Steiner, Sharon M. Donovan, Ryan N. Dilger

**Affiliations:** ^1^Piglet Nutrition and Cognition Laboratory, Department of Animal Sciences, University of Illinois at Urbana-Champaign, Urbana, IL, United States; ^2^Neuroscience Program, University of Illinois at Urbana-Champaign, Urbana, IL, United States; ^3^Nestlé Research, Lausanne, Switzerland; ^4^Nestlé Product Technology Center Nutrition, Vevey, Switzerland; ^5^Department of Food Science and Human Nutrition, University of Illinois at Urbana-Champaign, Urbana, IL, United States; ^6^Division of Nutritional Sciences, University of Illinois at Urbana-Champaign, Urbana, IL, United States

**Keywords:** prebiotics, milk, pig, brain, nutrition, cognition, neuroimaging, human milk oligosaccharides (HMOs)

## Abstract

Human milk contains a unique profile of oligosaccharides (OS) and preliminary evidence suggests they impact brain development. The objective of this study was to assess the impact of bovine and/or human milk oligosaccharides (HMO) (2′-fucosyllactose and Lacto-N-neotetraose) on cognition, brain development, and hippocampal gene expression. Beginning on postnatal day (PND) 2, male pigs received one of four milk replacers containing bovine milk oligosaccharides (BMOS), HMO, both (BMOS + HMO), or neither. Pigs were tested on the novel object recognition task using delays of 1- or 48-h at PND 22. At PND 32–33, magnetic resonance imaging procedures were used to assess structural brain development and hippocampal tissue was collected for analysis of mRNA expression. Pigs consuming only HMO exhibited recognition memory after a 1-h delay and those consuming BMOS + HMO exhibited recognition memory after a 48-h delay. Both absolute and relative volumes of cortical and subcortical brain regions were altered by diet. Hippocampal mRNA expression of *GABRB2*, *SLC1A7*, *CHRM3*, and *GLRA4* were most strongly affected by diet. HMO and BMOS had distinct effects on brain structure and cognitive performance. These data suggest different mechanisms underlie their influence on brain development.

## Introduction

Human milk oligosaccharides (HMO) are increasingly recognized as important promoters of intestinal and immune development (Kirmiz et al., 2018). Human milk oligosaccharides are the third most concentrated solid in human milk behind lactose and lipids (Bode, 2012), and their diversity and concentration are unlike any other species (Urashima et al., 2001). Although bovine milk contains OS that are structurally similar or identical to those found in human milk, they are found in lower concentrations than in human milk (Gopal and Gill, 2000; Martín-Sosa et al., 2003). Emerging research has suggested that bovine milk derived oligosaccharides (BMOS) and HMO [e.g., 2′-fucosyllactose (2′-FL), Lacto-N-neotetraose (LNnT)] may confer physiological benefits when consumed by formula-fed infants.

Infants fed formula supplemented with BMOS have shown tolerance and adequate growth as measured by anthropomorphic measures (e.g., weight-for-age, length-for-age, or head-circumference scores) and stool characteristic (e.g., frequency, consistency, or pH) closer to that of breastfed infants (Meli et al., 2014; Cooper et al., 2016; Simeoni et al., 2016; Radke et al., 2017). Provision of a combination of BMOS and *Bifidobacterium animalis* subsp. *Lactis* brought fecal bifidobacteria counts of infants delivered via C-section closer to that of infants delivered vaginally (Cooper et al., 2016), and the same combination brought 16S ribosomal RNA sequencing indices of richness, evenness, and the Shannon index similar to that of a breastfed control group.

Infants fed formula supplemented with HMO demonstrated age appropriate growth (Marriage et al., 2015; Puccio et al., 2017), fewer reports of respiratory illness or use of antibiotics/antipyretics (Puccio et al., 2017), and stool consistency and frequency closer to that of breastfed infants (Marriage et al., 2015). Absorption and excretion of 2′-FL is consistent with the amount of 2′-FL consumed, with breastfed and 2′-FL supplemented infants displaying higher concentrations of plasma and urinary 2′-FL (Marriage et al., 2015). Furthermore, infants fed formula containing 2′-FL in addition to galactooligosaccharide (GOS) demonstrated plasma cytokine (e.g., IL-1ra, TNF-α, IL-1α, IL-1β, IL-6) concentrations closer to that of breastfed infants than those consuming GOS only supplemented formulas. When peripheral blood mononuclear cells were stimulated with respiratory syncytial virus *ex vivo* the cytokine response from breastfed and 2′-FL supplemented infants were similar (Goehring et al., 2016).

Recent reports demonstrate that various oligosaccharides are also capable of promoting brain development (Kao et al., 2016). Rats supplemented with 2′-FL demonstrated vagally mediated improved learning and memory (Vázquez et al., 2015, 2016; Oliveros et al., 2016), rats supplemented with sialyllactose demonstrated improved behavioral response to stress (Tarr et al., 2015), and we have demonstrated that pigs fed a combination of polydextrose and GOS (Fleming et al., 2017) have improved performance in the novel object recognition (NOR) task. Various oligosaccharides have separately proven capable of altering brain or cognitive development in some manner, however few studies have directly compared the efficacy of BMOS and HMO.

Therefore, the objective of the present study was to assess the efficacy of BMOS and HMO alone or in combination at altering performance in the NOR task, structural neurodevelopment, or hippocampal mRNA expression in the young pig.

## Materials and Methods

### Animals and Housing

All animal care and experimental procedures were in accordance with the National Research Council Guide for Care and Use of Laboratory Animals and approved by the University of Illinois at Urbana-Champaign Institutional Animal Care and Use Committee. Housing and rearing protocols were adapted from previous studies in our lab (Fleming et al., 2017; Mudd et al., 2018), and are described as follows. Forty-eight intact male pigs (1050 Cambro genetics) were naturally farrowed and allowed colostrum consumption for up to 48 h before transport to the Piglet Nutrition & Cognition Laboratory at the University of Illinois at Urbana-Champaign. Pigs were artificially reared from postnatal day (PND) 2 until PND 33. This study was conducted using six independent cohorts (*n* = 2 pigs per dietary treatment in each cohort), with litter and initial bodyweight counterbalanced between dietary groups and within each cohort. All pigs were housed in master caging units that contained six individual stainless-steel cages (L × W × H of 87.6 cm × 88.9 cm × 50.8 cm) with clear, polycarbonate facades on three sides of the cage and vinyl-coated, expanded-metal flooring (Tenderfoot^®^, Minneapolis, MN, United States). The master unit was designed such that there were three separate levels each with two individual pig cages on each level. Pigs on each level shared a common wall containing holes to permit pigs to see, smell, hear, and minimally touch one-another. A towel and toy were included in each cage to provide enrichment, all pigs were removed from cages and allowed to socialize with each other for approximately 30 min each day, and all pigs were allowed *ad libitum* access to water at all times.

**FIGURE 1:**
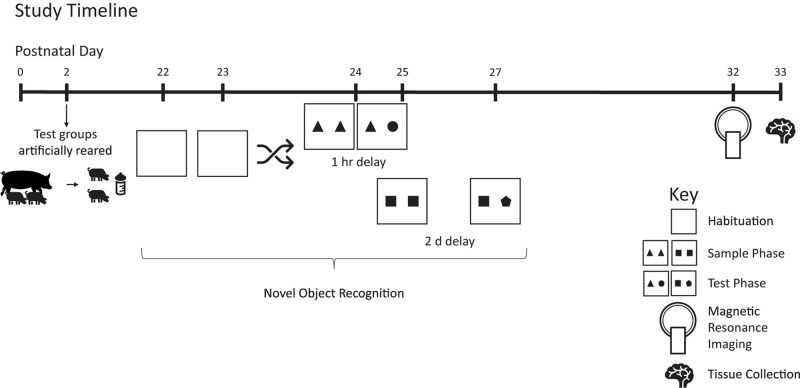
Study timeline. Pigs were reared artificially from postnatal days 2–33. Starting on postnatal day 22, pigs were tested on the novel object recognition test twice using delays of 1 h or 2 days, with task order randomized and counterbalanced between groups. On PND 32–33, pigs were subjected to magnetic resonance imaging and on PND 33 brain tissue was collected for quantification of hippocampal gene expression. Pigs were weighed daily to track growth.

All pigs were reared in the same room with ambient temperature maintained between 27 and 29°C and a 12-h light/dark cycle maintained from 0600 to 1800 h. Prior to placement in the artificial rearing system, pigs were administered 5 mL of *Clostridium perfingens* antitoxin C + D per the manufacturer’s recommendations (Colorado Serum Company, Denver, CO, United States) to prevent enterotoxemia (Niilo, 1988). At study conclusion (PND 33), pigs were anesthetized using a telazol:ketamine:xylazine solution (50 mg tiletamine plus 50 mg of zolazepam reconstituted with 2.50 mL ketamine [100 g/L] and 2.50 mL xylazine [100 g/L]; Fort Dodge Animal Health) by intramuscular injection at 0.03 mL/kg bodyweight. After anesthetic induction, pigs were euthanized via intracardiac administration of sodium pentobarbital (86.0 mg/kg of body weight; Euthasol, Virbac Animal Health, Fort Worth, TX). Two pigs were removed from study due to failure-to-thrive (diet: BMOS + HMO). See Figure 1 for an overview of the study design.

### Dietary Treatments

All researchers involved with conducting the study and acquiring and analyzing study results remained blind to dietary treatment identity until final data analyses were complete. Pigs (*n* = 12 per diet) were provided milk replacers reconstituted at 200 g of dry powder per 800 g of water. Reconstituted diets were formulated to contain approximately 0 g/L milk OS (control [CON], ProNurse^®^ Specialty Milk Replacer, Purina Animal Nutrition, Gray Summit, MO), 12.4 g/L BMOS (BMOS, Nestlé Product & Technology Center, Konolfingen, Switzerland), 1.5 g/L HMO (HMO, 1.0 g/L of 2′-FL + 0.5 g/L of LNnT, Glycom, Hørsholm, Denmark), or both bovine and human milk oligosaccharides (BMOS + HMO; 12.4 g/L of BMOS + 1.0 g/L of 2′-FL + 0.5 g/L of LNnT). Briefly, the BMOS product was derived from bovine whey and composed primarily of GOS and trace amounts of 3′ and 6′ sialyllactose, and is the same as that used in previous clinical trials (Meli et al., 2014; Cooper et al., 2016; Radke et al., 2017). Doses of each type of OS were not equal across treatment groups and were chosen to replicate those used in previous clinical studies where infants were provided formula containing 0.2 or 1 g/L 2′-FL (Marriage et al., 2015; Goehring et al., 2016; Puccio et al., 2017), 0.5 g/L LNnT (Puccio et al., 2017), 8 g/L BMOS (Cooper et al., 2016; Radke et al., 2017), or 10 g/L BMOS (Meli et al., 2014). Analyzed concentrations of each OS demonstrated success in replicating the concentration of HMO from previous clinical trials, with BMOS at approximately half the concentration used as compared to clinical research (5.8 g/L vs 8–10 g/L). All diets were formulated to contain lactose such that each diet provided the same amount of total carbohydrate. The nutritional composition of the base formula can be found in Table 1, and *post hoc* analysis of the oligosaccharide content of the formulated diets is shown in Table 2.

**TABLE 1:** Formulated nutrient composition of the base formula^1,2^.

		**Base formula**		**Final composition**^**3**^	
**Nutrient**	**Units**	**Per kg**	**Per Liter**	**Per kg**	**Per Liter**
**Energy and macronutrients**					
Metabolizable energy	kcal	4286.4	857.3	3989.4	797.9
Crude protein	g	241.0	48.2	224.3	44.9
Crude fat	g	241.0	48.2	224.3	44.9
Lactose	g	369.1	73.8	343.5	68.7
Crude fiber	mg	20.0	4.0	18.6	3.7
Ash	g	85.3	17.1	79.4	15.9
**Minerals**					
Calcium	mg	1000.0	200.0	930.7	186.1
Copper	mg	12.1	2.4	11.2	2.2
Total phosphorous	mg	800.0	160.0	744.6	148.9
Potassium	mg	1835.0	367.0	1707.8	341.6
Selenium	μg	875.0	175.0	814.4	162.9
Sodium	mg	345.0	69.0	321.1	64.2
Zinc	mg	120.0	24.0	111.7	22.3
**Vitamins and other nutrients**					
Vitamin A	IU	82427.3	16485.5	76715.0	15343.0
Vitamin D	IU	11563.9	2312.8	10762.5	2152.5
Vitamin E	IU	253.3	50.7	235.7	47.1
Lysine	g	25.2	5.0	23.4	4.7
Methionine + Cysteine	g	9.9	2.0	9.2	1.8

*^1^ProNurse^®^ Specialty Milk Replacer (Purina Animal Nutrition, Gray Summit, MO) was used as the base formula and nutritional composition is adapted from advertised nutrient composition. ^2^Milk was reconstituted at a rate of 200 g of milk replacer per 800 g of water. ^3^The base formula was diluted 6.93% to allow addition of oligosaccharides.*

**TABLE 2:** Carbohydrate content of the diets^1^.

	**Bovine milk oligosaccharides**^**2**^		**2’-Fucosyllactose**^**3**^		**Lacto-N-neotetraose**^**3**^		**Additional lactose**		**Total oligosaccharide**		**Total lactose**		**Total carbohydrate**	
**Diet**	**Per kg**	**Per Liter**	**Per kg**	**Per Liter**	**Per kg**	**Per Liter**	**Per kg**	**Per Liter**	**Per kg**	**Per Liter**	**Per kg**	**Per Liter**	**Per kg**	**Per Liter**
**Formulated**														
CON	0.00	0.00	0.00	0.00	0.00	0.00	69.30	13.86	0.00	0.00	412.79	82.56	412.79	82.56
BMOS	61.98	12.40	0.00	0.00	0.00	0.00	61.98	1.47	61.98	12.40	350.82	70.16	412.79	82.56
HMO	0.00	0.00	4.92	0.98	2.41	0.48	7.33	12.40	7.33	1.47	405.47	81.09	412.79	82.56
BMOS + HMO	61.98	12.40	4.92	0.98	2.41	0.48	0.00	0.00	69.30	13.86	343.49	68.70	412.79	82.56
**Analyzed**														
CON	0.00	0.00	0.00	0.00	0.00	0.00	NQ	NQ	0.00	0.00	NQ	NQ	NQ	NQ
BMOS	28.95	5.79	0.00	0.00	0.00	0.00	NQ	NQ	28.95	5.79	NQ	NQ	NQ	NQ
HMO	0.00	0.00	4.05	0.81	2.10	0.42	NQ	NQ	6.15	1.23	NQ	NQ	NQ	NQ
BMOS + HMO	28.75	5.75	5.00	1.00	2.65	0.53	NQ	NQ	36.40	7.28	NQ	NQ	NQ	NQ

*^1^Abbreviations: CON, control group; HMO, pigs fed human milk oligosaccharides; BMOS; pigs fed bovine milk oligosaccharides, BMOS + HMO, pigs fed both human and bovine milk oligosaccharides; NQ, not quantified. ^2^Nestlé Product & Technology center, Konolfingen, Switzerland. ^3^Glycom, Hørsholm, Denmark.*

Pigs received small volumes (approximately 500 mL) of experimental diets on the day of arrival to the rearing facility to allow for adjustment to the milk replacer prior to the standard feeding regimen. Pigs were fed at a rate of 285 and 325 mL of reconstituted diet per kg bodyweight from PND 3–6 and PND 7–33, respectively. Individual pig bodyweight was recorded daily to determine the volume of milk to be dispensed to individual animals throughout the day. Meals were administered 10 times a day, approximately every 100 min, between 1000 and 0400 h using an automated feeding system. Feed refusals were not quantified.

### Behavior

Pigs were tested on the NOR task using two different delays to assess intermediate and long-term recognition memory. Methods used were adapted from previous studies using this task in pigs from other labs (Moustgaard et al., 2002; Gifford et al., 2007; Kornum et al., 2007; Kouwenberg et al., 2009) and our own (Fleming and Dilger, 2017; Fleming et al., 2017, 2018). Testing consisted of a habituation phase, a sample phase, and a test phase. During the habituation phase, each pig was placed in an empty testing arena for 10 min each day for two days leading up to the sample phase. In the sample phase, the pig was placed in the arena containing two identical objects and given 5 min for exploration. After a delay of 1- or 48-h the pig was returned to the arena for the test phase of the NOR task. During the test phase, the pig was placed in the arena containing one object from the sample phase and a novel object and allowed to explore for 5 min. Between trials, objects were removed, immersed in hot water with detergent, and rubbed with a towel to mitigate odor and the arena was sprayed with water to remove urine and feces. Objects chosen had a range of characteristics (i.e., color, texture, shape, and size), however the novel and sample objects only differed in shape and size. Only objects previously shown to elicit a null preference were used for testing (Fleming and Dilger, 2017). Task order was counterbalanced between replicates. Habituation trials began at PND 22 and testing on the sample phase began on PND 24. Recognition index, or the proportion of time spent with the novel object compared to total exploration of both objects, was used to measure recognition memory. A recognition index significantly above 0.50 demonstrates a novelty preference and thus recognition memory. Videos from all experiments were analyzed using a commercially available software package (Ethovision XT 11^®^, Noldus Information Technology, Wageningen, Netherlands). Time spent investigating objects was recorded manually by mapping start and stop conditions to specific keys on a computer keyboard and total movement was assessed by tracking center-of-mass of the pig, including during habituation trials. Experimenters were blind to all treatment conditions during analysis. Investigations were classified as nose directed behavior such as rooting, mouthing, or sniffing of the objects. Rubbing up against, standing over, standing near, looking at, or sniffing the floor/air near the objects were not counted as investigations.

### Magnetic Resonance Imaging

All pigs underwent magnetic resonance imaging (MRI) procedures at PND 32 or 33 at the Beckman Institute for Advanced Science and Technology Biomedical Imaging Center using Siemens MAGNETOM Trio 3T equipment with a Siemens 32-channel head coil. Methods described here are adapted from previous studies using MRI in pig (Radlowski et al., 2014; Jacob et al., 2016; Mudd et al., 2016b). Each pig underwent imaging protocols only once. The pig neuroimaging protocol included three magnetization prepared rapid gradient-echo (MPRAGE) sequences and diffusion tensor imaging (DTI) to assess brain macrostructure and microstructure, respectively, as well as magnetic resonance spectroscopy (MRS) to obtain brain metabolite concentrations. In preparation for MRI procedures, anesthesia was induced using an intramuscular injection of telazol (50.0 mg of tiletamine plus 50.0 mg of zolazepam reconstituted with 5.0 DI water; Zoetis, Florham Park, NJ) administered at 0.07 mL/kg bodyweight, and maintained with inhalation of isoflurane (98% O_2_, 2% isoflurane). Pigs were immobilized during all MRI procedures. Visual observation of each pig’s well-being, as well as observations of heart rate, PO_2_ and percent of isoflurane were recorded every 5 min during the procedure and every 10 min post-procedure until animals recovered. Total scan time for each pig was approximately 60 min. Imaging techniques are briefly described below.

#### Structural MRI

A T_1_-weighted MPRAGE sequence was used to obtain anatomic images of the pig brain with a 0.7 mm isotropic voxel size. Three repetitions were acquired and averaged using SPM8 in Matlab 8.3, and brains were manually extracted using FMRIB Software Library (FSL; FMRIB Centre, Oxford, United Kingdom). The following sequence specific parameters were used to acquire T_1_-weighted MPRAGE data: repetition time (TR) = 1900 ms; echo time (TE) = 2.49 ms; 224 slices; field of view (FOV) = 180 mm; flip angle = 9°. Methods for MPRAGE averaging and manual brain extraction were previously described (Mudd et al., 2016b). All data generated used a publicly available population-averaged pig brain atlas^1^ (Conrad et al., 2014). For volumetric assessments, individual brains were segmented into 23 regions of interest (ROI) using the pig brain atlas. Total brain and individual region volume analysis was performed in which an inverse warp file for each ROI was generated from the DARTEL-generated warp files for each region. As described previously (Mudd et al., 2016b), the SPM “Segment” tool, along with pig specific tissue prior probabilities, was used to obtain gray matter, white matter, and cerebrospinal fluid (CSF) tissue segmentations for each pig, and DARTEL was used to align the native space segmentations. The fslstats toolbox was used to determine the voxel volume of the subject-space segmentation for each of the three tissue types. Using fslmaths, the mean overall partial volume map was obtained for each subject-space segmentation. Overall absolute volume for gray matter, white matter, and CSF was determined by multiplying the voxel volume measure by the mean intensity of the partial volume segmentation. In order to account for absolute whole-brain volume, all ROI were also expressed as a percent of total brain volume (%TBV).

#### Diffusion Tensor Imaging

Diffusion tensor imaging was used to assess white matter maturation and axonal tract integrity using a *b*-value = 1000 s/mm^2^ across 30 directions and a 2 mm isotropic voxel. Diffusion-weighted echoplanar images (EPI) were assessed in FSL 5.0 for fractional anisotropy (FA), mean diffusivity (MD), axial diffusivity (AD), and radial diffusivity (RD) using methods previously described (Mudd et al., 2016b). The pig brain atlas was used for assessment of the following ROI: caudate, corpus callosum, cerebellum, both hippocampi, internal capsule, left and right cortex, thalamus, DTI-generated white matter, and atlas-generated white matter was performed using a customized pig analysis pipeline and the FSL software package. The diffusion toolbox in FSL was used to generate values of AD, RD, MD, and FA. In the corresponding results, atlas-generated white matter indicates the use of white matter prior to using probability maps from the pig brain Fatlas that were used as a region of interest mask. Likewise, DTI-generated white matter indicates a threshold of 0.2 was applied to FA values, restricting analysis to white matter tracts. Masks for each ROI from the atlas were non-linearly transformed into the MPRAGE space of each individual pig and a linear transform was then applied to transfer each ROI into DTI space. A threshold of 0.5 was applied to each ROI, and the data were dilated twice. For each individual ROI, an FA threshold of 0.15 was applied to ensure that we included only white matter in that region of interest despite the mask expansion.

#### Magnetic Resonance Spectroscopy

Magnetic resonance spectroscopy was used to non-invasively quantify metabolites in the whole brain. The MRS spin-echo chemical shift sequence was used with a voxel size of 20 mm × 23 mm × 13 mm and centered over the left and right dorsal hippocampi. The following sequence parameters were used in acquisition of spectroscopy data for the water-suppressed scan: TR = 1800 ms; TE = 68 ms; signal averages = 256, vector size = 1024. The following sequence parameters were used in acquisition of spectroscopy data for the non-water-suppressed scan: TR = 20,000 ms; TE = 68 ms; signal averages = 1; vector size = 1024 point. Both water-suppressed and non-water-suppressed data were collected in institutional units, and all MRS data were analyzed with LC Model (version 6.3) using methods previously described (Radlowski et al., 2014). Limits were placed on MRS data for inclusion in the statistical analysis. Cramer-Rao lower bounds (i.e.,% standard deviation) were calculated using LC Model and only metabolites with a standard deviation less than 20% were considered to have reliable quantitative results of absolute levels.

### Hippocampal Gene Expression

Approximately 20 mg of hippocampal tissue were introduced in a Lysing Matrix D tube (MP Biomedicals, Santa Ana, CA, United States), placed on ice, and 650 μL of lysis buffer (Agencourt RNAdvance Tissue Kit, Beckman Coulter, Indianapolis, IN, United States) was added. Tubes were agitated for 2 × 1 min at speed 6 on FastPrep^®^-24 (MP Biomedicals, Santa Ana, CA, United States), and 400 μL of lysate were then extracted using the Agencourt RNAdvance Tissue Kit (Beckman Coulter, Indianapolis, IN, United States) following the manufacturer’s recommendations. RNA were quantified using the Quant-iT^TM^ RiboGreen^TM^ RNA Assay Kit (Invitrogen, Carlsbad, CA, United States) on a Spectramax M2 (Molecular Devices, Sunnyvale, CA, United States). RNA quality assessment was completed using a Fragment Analyzer 96 with Standard Sensitivity RNA Analysis Kit (15 nt) (Advanced Analytical Technologies, Inc., Ankeny, IW, United States). Relative mRNA copy number on 93 genes was quantified using the NanoString nCounter^TM^ system (NanoString Technologies Inc., Seattle, WA, United States) according to the manufacturer’s instructions using 100ng of RNA as the starting amount. Using nSolver software (Version 4.0, NanoString Technologies Inc., Seattle, WA, United States), background subtraction using the median of all eight negative controls was followed by positive control normalization using the geometric mean of six positive controls and housekeeping normalization using the geometric mean of six housekeeping genes.

### Statistical Analysis

Data analysis was conducted using the GLIMMIX procedure of SAS Enterprise Guide 7.1 (SAS Institute, Cary, NC, United States). All data were subjected to a two-way analysis of variance to assess the main effects of BMOS, HMO, and the interaction effect of BMOS and HMO. Two animals were removed from study (both provided BMOS + HMO). One pig was removed from study at PND 2 due to poor growth and inability to gain weight. The second pig was removed due to unexpected mortality after behavioral assessment and prior to MRI acquisition, however behavioral data was included in the analysis (all behavioral and growth measures were within normal ranges). Cohort of pig was included in the model as a random variable. For all variables, observations with a studentized residual greater than | 3| were considered outliers and removed from that variable only. For behavioral data, pigs that exhibited little exploration of either object (i.e., less than 2 s of exploration of either the sample or novel object) were considered non-compliant and their recognition index was not measured in the test phase (total sample sizes of 1-h delay: CON, *n* = 9; BMOS, *n* = 10; HMO, *n* = 10; BMOS + HMO, *n* = 11. 2-day delay: CON, *n* = 10; BMOS, *n* = 9; HMO, *n* = 11; BMOS + HMO, *n* = 10), but all other exploration measures were included for those subjects. This criteria was chosen to prevent the analysis of pigs that tend to “perseverate” on singular objects while ignoring the exploration of the rest of the arena or other objects, while requiring all animals analyzed to have engaged in a minimal amount of investigation of both objects. Variables from the sample phases from the 1- and 48-h delay paradigms were averaged to create sample phase exploration measures (e.g., total time visiting objects during the sample phase in the 1- and 48-h delay were average to create a single measure). To test for recognition memory, a one-sample t-test was conducted comparing the recognition index to a null mean of 0.5. Groups with a mean recognition index significantly above 0.5 as measured by a right-tailed one-sample t-test were considered to demonstrate recognition memory.

For individual brain region volume assessment, volume was expressed in both absolute (i.e., mm^3^) and relative units (i.e., regional volume as a proportion of total brain volume, within subject). Gene expression data were standardized (mean of zero and standard deviation of one) and centered by the control group, thus all z-scores for the control group are zero. Statistical significance was defined at *P* < 0.05 (insignificant results provided in Supplementary Tables). *Post hoc* comparisons for mean separation were conducted with a Tukey adjustment, and data are represented as least square means. Correlations between significant outcomes (MRI or gene expression) gene expression and the recognition index were conducted using the Pearson correlation coefficient for each diet and linear regression was used to assess the diet independent relationship between outcomes. Sample sizes for all variables assessed can be found in the Supplementary Tables.

## Results

### Growth and Behavior

**FIGURE 2:**
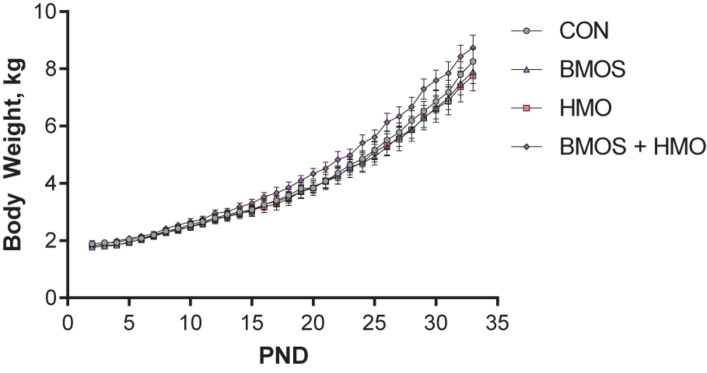
Body weight (BW) during the trial. No differences in average daily body weight gain were observed between groups (*P* = 0.15).

**FIGURE 3:**
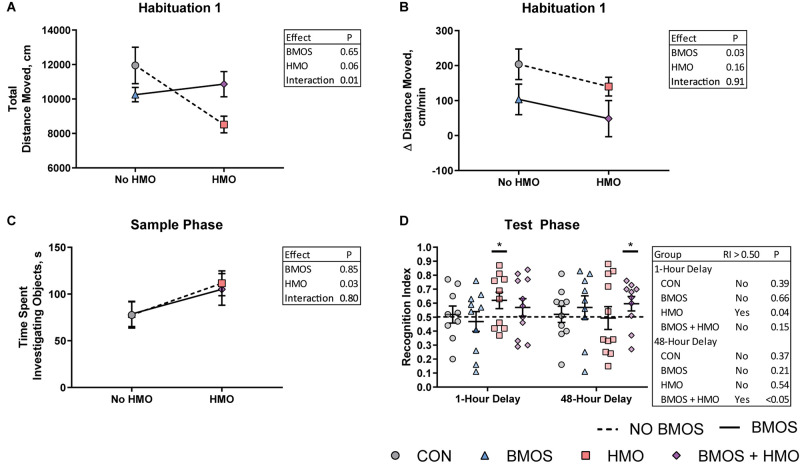
Performance during the novel object recognition task assessed using a two-way analysis of variance including BMOS, HMO, and their interaction. **(A)** An interaction effect was observed for total distance moved during the first habituation trial. **(B)** During the first habituation trial, pigs fed diets containing BMOS showed a lesser increase in distance moved per minute than those not fed BMOS. **(C)** During the sample phase pigs fed HMO in any diet spent more time investigating objects than those fed diets without HMO. **(D)** Pigs fed the HMO-only diet were able to display a recognition index greater than 0.5 after a 1-h delay, however only those fed both BMOS and HMO were able to display a recognition index greater than 0.5 after a 48-h delay, as indicated by the asterisks (measured by a one-sample *t*-test for a recognition index greater than 0.5).

Daily body weights were nearly equivalent between groups. Despite the BMOS + HMO group appearing to separate from the other groups, there was no difference in average daily body weight gain between groups over the course of the study (*P* = 0.15, Figure 2). Though milk intake was not quantified, equivalent growth suggests pigs consumed similar amounts of milk replacer per day as milk was dosed according to body weight. During the first habituation trial, pigs fed only HMO displayed less total movement in the arena as compared to controls (*P* = 0.008, Figure 3A). Pigs fed any diet containing BMOS (as BMOS or BMOS + HMO) displayed a lower increase in distance moved per minute across the duration of the first habituation trial than those not fed BMOS (*P* = 0.027, Figure 3B). There were no significant differences in any of the scored behaviors during the second habituation trial between groups (all *P* ≥ 0.223). During the sample phase pigs fed diets containing HMO (as HMO or BMOS + HMO) spent more time investigating objects than those not fed diets containing HMO (*P* = 0.032, Figure 3C). The HMO group was able to show recognition memory after a 1-h delay (one-sample *t*-test, *P* = 0.038, Figure 3D), whereas only the BMOS + HMO group displayed recognition memory after a 48-h delay (one-sample *t*-test, *P* = 0.045, Figure 3D). Despite these differences, exploratory behavior (e.g., time spent investigating or number of visits to the sample or novel object) during the test phase was not significantly different between groups (all *P* ≥ 0.053, Supplementary Table 1).

### Magnetic Resonance Imaging

**FIGURE 4:**
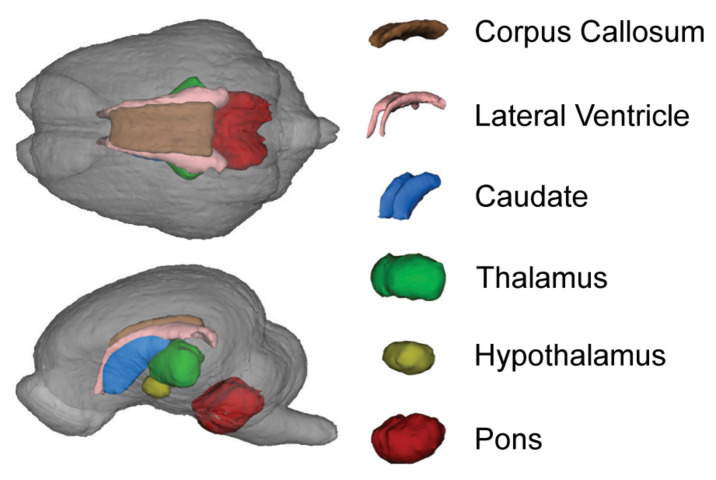
Representative 3D surface rendering of the brain from the pig brain atlas. Only brain regions altered by the diet are shown. Although affected by diet, the cortices and gray matter are not highlighted to allow visualization of subcortical structures.

**FIGURE 5:**
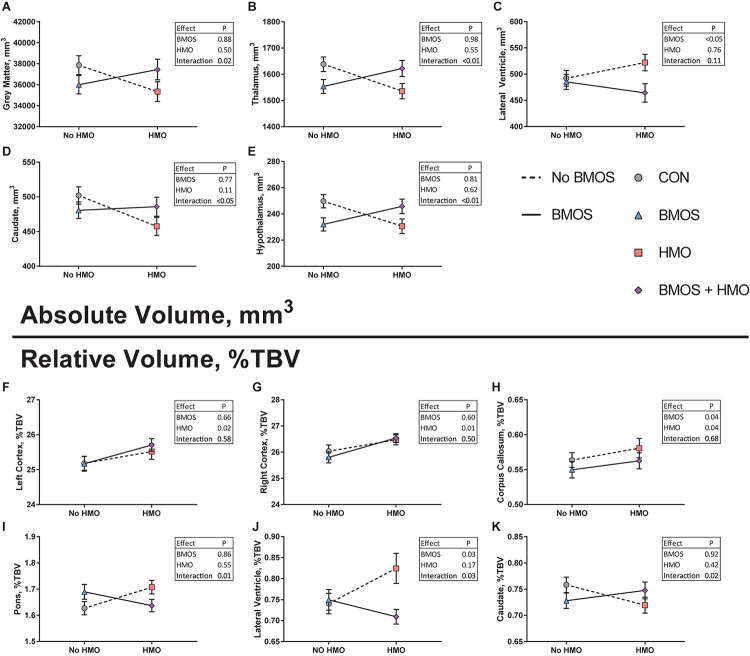
**(A–E)** Absolute volume of regions affected by diet assessed using a two-way analysis of variance including BMOS, HMO, and their interaction. In each case, (except **C)** an interaction effect was observed wherein pigs fed the CON or BMOS + HMO diet displayed similar absolute volumes compared to pigs fed either BMOS or HMO. **(F–K)** Relative volumes of regions affected by diet. **(F,G)** A main effect of HMO was seen for both the left and right cortices, pigs fed any diet containing HMO demonstrating larger relative cortices. **(H)** Pigs fed diets containing HMO had a larger relative corpus callosum, whereas pigs fed diets containing BMO had a smaller relative corpus callosum. **(I–K)** Similar to the effects seen for absolute volumes, interactions effects of diet for relative volumes of the caudate, lateral ventricle, and pons were observed, wherein pigs fed the CON or BMOS + HMO diet displayed similar relative volumes compared to pigs fed either BMOS or HMO.

A 3D surface rendering of the brain regions affected by the diet is shown in Figure 4. Interaction effects of BMOS and HMO were observed for absolute volumes of the caudate, hypothalamus, thalamus, and total gray matter, and a main effect of BMOS was observed for the lateral ventricle (All *P* ≤ 0.047, Supplementary Table 2, Figures 5A–E). For all interaction effects observed for absolute volumes, pigs provided HMO demonstrated larger volumes of these regions if the diet also contained BMOS, whereas those consuming diets without HMO demonstrated smaller volumes if the diet also contained BMOS (Figures 5A,B,D,E). As a proportion of total brain volume, interaction effects were observed for the caudate, lateral ventricle, and pons, with main effects of BMOS or HMO for the corpus callosum, lateral ventricle, left cortex, and right cortex (all *P* ≤ 0.042, Supplementary Table 3, Figures 5F–K). There was no impact of diet for any DTI measure (AD, MD, RD, or FA, all *P* ≥ 0.063, Supplementary Table 4). Of the MRS outcomes, only glutathione, myo-inositol, N-acetylaspartate, and γ-amino butyric acid met criteria for inclusion, however none were altered by diet (all *P* ≥ 0.057, Supplementary Table 5).

### Hippocampal Gene Expression

**FIGURE 6:**
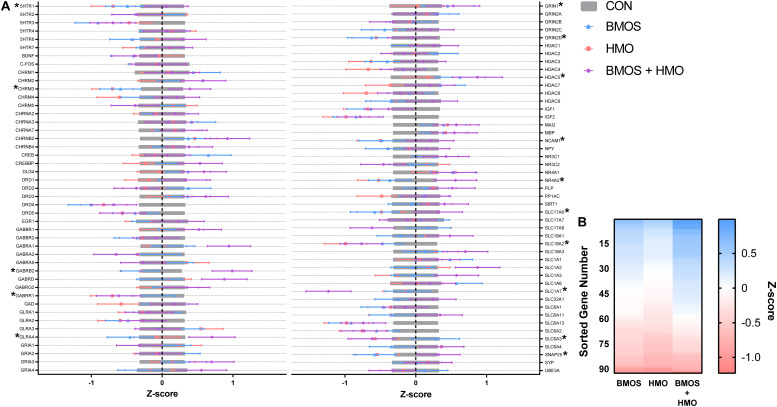
**(A)** Hippocampal tissue was assessed for the mRNA expression of 93 genes. Figure depicts standardized data (mean = 0, standard deviation = 1) centered by control group. Values below zero indicate decreased expression compared to control, whereas values above zero indicate increased expression. Bars show mean ± standard error, genes significantly impacted by diet are denoted by an asterisk. Accession numbers for each gene can be found in Supplementary Table 6. **(B)** Genes were sorted in descending order by Z-score for each diet, visualizing the trend where the HMO group exhibited greater downregulation of mRNA compared to the BMOS or BMOS + HMO group. CON, control group; BMOS, pigs fed a mixture of bovine milk oligosaccharides; HMO, Pigs fed 2’-fucosyllactose + Lacto-*N*-neotetraose; BMOS + HMO, pigs fed both human and bovine milk oligosaccharides.

No samples were found outside the normalization factor ranges for positive controls (0.3–3.0) or housekeeping genes (0.1–10). Some samples demonstrated mRNA counts of some genes below the threshold for background subtraction, and these are detailed in Table 3 and Supplementary Table 6. Numerous effects of diet were observed for measures of hippocampal mRNA expression. Outcomes affected included: serotonin receptor 1 (*5HTR1*), the cholinergic receptor muscarinic 3 (*CHRM3*), the GABA type A receptor subunit β2 (*GABRB2*), the GABA type A receptor subunit ρ1 (*GABRR1*), the glycine receptor α4 (*GLRA4*), the glutamate ionotropic receptor NMDA type subunit 1 and subunit 2D (*GRIN1* and *GRIN2D*), the histone deacetylase 5 (*HDAC5*), the neural cell adhesion molecule 1 (*NCAM1*), the Nuclear Receptor Subfamily 4 Group A Member 2 (*NR4A2*), the Solute Carrier Family 17 Member 6 (*SLC17A6*), the Solute Carrier Family 18 Member A2 (*SLC18A2*), the Solute Carrier Family 1 Member 7 (*SLC1A7*), the Solute Carrier Family 6 Member 3 (*SLC6A3*), and the synaptosome associated protein 25 (*SNAP25*) (all *P* ≤ 0.048). Both BMOS and HMO groups showed downregulation of many genes compared to controls, whereas pigs fed BMOS + HMO displayed upregulation of those same genes. See Table 3 and Figure 6A for a description of the effects observed. After a Tukey adjustment for multiple comparisons, only *CHRM3*, *GABRB2*, *GLRA4*, and *SLC1A7* were significantly altered by diet (Table 3). To visualize the general trend for mRNA to be up- or downregulated as compared to controls, within diet, genes were ordered by descending average Z-score and plotted on a heatmap (Figure 6B). Results for all genes measured can be found in Supplementary Table 6.

**TABLE 3:** Standardized mRNA expression of genes affected by dietary treatment^1^.

**Measure**^3^	**CON**	**BMOS**	**HMO**	**BMOS + HMO**	**Pooled**	***P*-Value**^**2**^		
					**SEM**	**BMOS**	**HMO**	**INT**
*5HTR1*^4^	0^a^	−0.49^a^	−0.69^a^	0.06^a^	0.31	0.654	0.808	0.039
*CHRM3*	0^ab^	−0.59^ab^	−0.70^b^	0.39^a^	0.29	0.366	0.612	0.004
*GABRB2*	0^ab^	−0.31^b^	−0.30^b^	0.99^a^	0.28	0.071	0.064	0.004
*GABRR1*^4^	0^a^	−0.12^a^	−0.70^a^	−0.61^a^	0.31	0.959	0.047	0.724
*GLRA4*^5^	0^ab^	−0.45^b^	−0.07^ab^	0.70^a^	0.32	0.548	0.048	0.027
*GRIN1*^4^	0^a^	0.45^a^	0.02^a^	0.53^a^	0.37	0.035	0.818	0.888
*GRIN2D*^4^	0^a^	−0.62^a^	−0.56^a^	−0.05^a^	0.34	0.822	0.979	0.037
*HDAC5*^4^	0^a^	0.62^a^	0.17^a^	0.87^a^	0.35	0.015	0.428	0.876
*NCAM1*^4^	0^a^	−0.48^a^	−0.50^a^	0.21^a^	0.33	0.682	0.743	0.041
*NR4A2*^4^	0^a^	−0.37^a^	−0.52^a^	0.56^a^	0.30	0.211	0.480	0.015
*SLC17A6*^4^	0^a^	−0.58^a^	−0.48^a^	0.32^a^	0.34	0.673	0.437	0.012
*SLC18A2*^4,5^	0^a^	−0.47^a^	−0.99^a^	−0.76^a^	0.30	0.670	0.031	0.230
*SLC1A7*	0^a^	−0.11^a^	−0.14^a^	−1.23^b^	0.32	0.026	0.020	0.066
*SLC6A3*^4,5^	0^a^	0.28^a^	−0.57^a^	−0.62^a^	0.34	0.689	0.018	0.593
*SNAP25*^4^	0^a^	−0.58^a^	−0.54^a^	0.32^a^	0.30	0.615	0.536	0.015

*^1^Abbreviations: CON, control group; HMO, pigs fed human milk oligosaccharides; BMOS; pigs fed bovine milk oligosaccharides, BMOS + HMO, pigs fed both human and bovine milk oligosaccharides; SEM, standard error of the mean; INT, interaction effect of HMO and BMOS; 5HTR1, serotonin receptor 1; CHRM3, cholinergic receptor muscarinic 3; GABRB2, GABA type A receptor subunit β2; GABRR1, GABA type a receptor subunit ρ1; GLRA4, glycine receptor α4; GRIN1 and GRIN2D, glutamate ionotropic receptor NMDA type subunit 1 and subunit 2D; HDAC5, histone deacetylase 5; NCAM1, the neural cell adhesion molecule 1; NR4A2, nuclear receptor subfamily 4 group a member 2; SLC17A6, solute carrier family 17 member 6; SLC18A2, solute carrier family 18 member A2; SLC1A7, solute carrier family 1 member 7; SLC6A3, solute carrier family 6 member 3; SNAP25, synaptosome associated protein 25. ^2^Data analyzed via two-way ANOVA with *post hoc* Tukey adjustment for multiple comparisons. ^3^Standardized values for mRNA expression (mean = 0, standard deviation = 1) centered by control group. Only measures significantly altered by diet are shown. ^4^Mean separation insignificant after Tukey adjustment. ^5^Number of samples below threshold (median of negative controls): *GLRA4*, 10; *SLC18A2*, 7; *SLC6A3*, 14. ^*abc*^Means without a common superscript differ (*P* < 0.05).*

### Correlation Analysis

**FIGURE 7:**
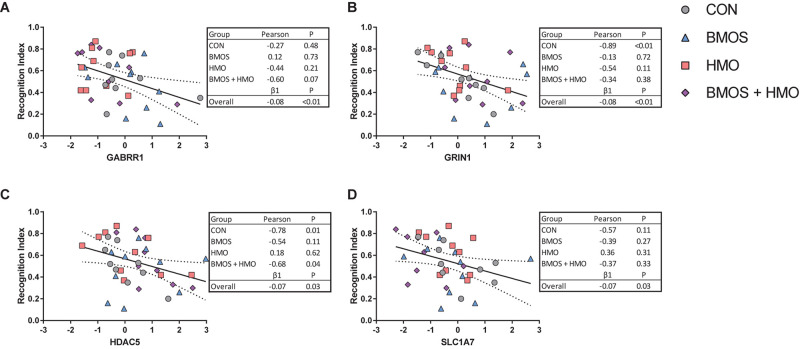
Of the variables affected by diet, the genes **(A–D)** *GABRR1*, *GRIN1*, *HDAC5*, and *SLC1A7* were shown to correlate with the recognition index after a 1-h delay. No relationships were found between MRI or gene expression outcomes affected by diet and the recognition index after a 48-h delay. The relationships observed were not present equally across all diets, however, study-wide all were negatively related to the recognition index.

To assess whether the outcomes were related or simply co-occurring, *post hoc* correlations and regression were conducted against the recognition indices from the 1 and 48-h delay trials against only those MRI and mRNA expression outcomes significantly affected by diet. A linear regression was conducted independent of diet (diet was not included in the model) to understand the overall trend between outcomes, and correlations were performed by diet group. Using the recognition index after a 1-h delay, four significant linear relationships were found between the recognition index and *GABRR1*, *GRIN1*, *HDAC5*, and *SLC1A7* (all *P* ≤ 0.028, Figure 7). There were no relationships between significant MRI outcomes and the recognition index on either delay (all *P* ≥ 0.12), and no relationships between significant gene expression outcomes and the recognition index after a 48-h delay (all *P* ≥ 0.11). Results for all correlations and regressions can be found in Supplementary Tables 7, 8.

## Discussion

The objective of this study was to assess the effect of early life dietary supplementation with HMO, BMOS, and their combination on promoting recognition memory, and altering brain structure and hippocampal mRNA expression. We found that while both BMOS and HMO are capable of affecting behavior, they appeared to do so in different ways. Furthermore, they had differential effects on both gene expression and MRI measures. Importantly, the study design was limited by the use of different doses for each source of oligosaccharide. However, this design was chosen to replicate the doses used in previous clinical trials (Meli et al., 2014; Marriage et al., 2015; Cooper et al., 2016; Goehring et al., 2016; Puccio et al., 2017; Radke et al., 2017). Provided this limitation and the inability to make a direct comparison between OS controlled for dose, the data suggest that OS are capable of affecting and improving behavior yet had less clear of an impact on MRI and gene expression measures. While several studies have examined the effect of single oligosaccharide supplementation on cognition and brain development (Schmidt et al., 2015; Vázquez et al., 2015, 2016; Jia et al., 2016; Savignac et al., 2016; Fleming et al., 2017; Yen et al., 2017), here we explored the individual and combined effects of BMOS and HMO.

The concentrations and diversity of OS in human milk vary to a great extent (McGuire et al., 2017). One of the most well studied OS, 2′-FL, is not even secreted in all women (Bode, 2012). Some broad species comparisons can still be made, however. The OS in human milk are primarily composed of neutral fucosylated OS, followed by neutral non-fucosylated, acidic (i.e., sialyllated), and acidic fucosylated OS (Kirmiz et al., 2018). Bovine milk is primarily composed of acidic and neutral non-fucosylated OS (Kirmiz et al., 2018). Although an oversimplification of their differences, bovine milk is largely absent in fucose containing OS and concentrated in sialic acid containing OS, whereas a significant portion of HMO contain fucose rather than sialic acid. Despite their differences in concentration, bovine milk does contain OS found in human milk [notably 3 or 6′-SL, 2 or 3′-FL, and LNnT (Aldredge et al., 2013)]. In the present study, the HMO provided were a combination of 2′-FL and LNnT, whereas the BMOS were predominantly a mixture of GOS, with small amounts of sialyllactose. Therefore, pigs fed the HMO diet consumed a diet with OS content similar to that of human milk (OS were either neutral fucosylated or neutral non-fucosylated), and those fed BMOS consumed a diet closer to that of bovine milk (mostly neutral non-fucosylated with small amounts of acidic OS).

Pigs consuming only HMO were able to display recognition memory after a 1-h delay, whereas only those pigs consuming both HMO and BMOS were able to display recognition memory after a 48-h delay. Pigs consuming the control diet were unable to display recognition memory on either the 1- or 48-h delay, however this has been typical of pigs not provided a prebiotic (Fleming et al., 2017). Although the effects on recognition memory are variable, pigs consuming any diet containing HMO displayed increased time investigating objects during the sample phase, clearly demonstrating that the presence of HMO in the diet increased object exploration.

Similar to behavior, consumption of HMO or BMOS did not produce consistent effects on the volume of various brain regions, rather the effects observed were specific to each brain region and varied between diets. On an absolute basis, a main effect of BMOS for the lateral ventricle and interaction effect of BMOS and HMO were observed for the gray matter, thalamus, caudate, and hypothalamus (Figures 5A–E). Relative to total brain volume, pigs fed any diet containing HMO demonstrated large volumes of the cortices and corpus callosum (Figures 5F–H). Additionally, a main effect of BMOS was found in the corpus callosum where BMOS-fed pigs demonstrated lower volume than pigs not fed BMOS (Figure 5H). There was an interaction of BMOS and HMO for the pons, lateral ventricle, and caudate. For the pons, supplementation with diets containing only BMOS or HMO increased the relative volume compared to controls and pigs fed BMOS + HMO, which demonstrated similar relative volumes (Figure 5I). In the lateral ventricle, both a main effect of BMOS and an interaction effect of BMOS + HMO were observed. Pigs fed diets containing HMO demonstrated the largest relative volume, with pigs fed BMOS + HMO the smallest (Figure 5J). Lastly, pigs fed BMOS + HMO demonstrated larger relative volumes of the caudate as compared to those fed only BMOS or HMO (Figure 5K).

Whether these changes are beneficial or detrimental is unclear, none of these changes appeared to be related to recognition memory when assessed via linear regression (Supplementary Tables 8, 9). Based on the absolute and relative volumes, the interaction effects show that the control and BMOS + HMO groups have similar values for almost all regions (with the exception of the lateral ventricle, cortices, and corpus callosum). Essentially, regional size was similar between pigs given no OS or both BMOS + HMO. An important confounder however is that the dosage of oligosaccharide was not controlled for. Therefore, pigs given HMO, BMOS, and BMOS + HMO consumed increasing levels of oligosaccharide, respectively. The pattern wherein pigs given HMO or BMO differ from those provided the control diet or HMO + BMOS may be indicative of a dose effect, however, this pattern is not evident in either behavioral or mRNA expression data. Past research from our lab has demonstrated that supplementation with sialyllactose had no effect on regional brain volumes but was able to alter the diffusivity of the corpus callosum and left hippocampus (Mudd et al., 2017). When supplemented with a mixture of prebiotics, lactoferrin, and milk fat globule membrane, the gray and white matter concentrations were most notably altered in the left and right cortex (Mudd et al., 2016a). Despite these changes, no differences were observed for spectroscopy or DTI in this study, suggesting that the diet did not affect white matter integrity or gross metabolic state.

When we examined hippocampal mRNA expression, intake of BMOS or HMO produced significant downregulation of several genes, whereas the intake of both BMOS and HMO produced upregulation in those same genes (of those shown in Table 3, *GABRR1, GRIN2D*, *SLC18A2*, *SLC1A7*, and *SLC6A3* did not follow this trend). Genes affected were related to many processes, however after a *post hoc* Tukey adjustment for multiple comparisons four genes were found most impacted by diet: *GABRB2*, *SLC1A7*, *GLRA4*, and *CHRM3*. As the genes affected represent several distinct processes (GABAergic, glutamatergic, and histone deacetylation) and were not affected by the diet in any clear pattern, we conducted a diet-independent linear regression to assess whether they were related to recognition memory. Interestingly, no gene examined correlated with recognition memory after a 48-h delay. Of all the genes affected shown in Table 3, four were significantly related to the recognition index after a 1-h delay: *GRIN1*, *GABBR1*, *HDAC5*, and *SLC1A7* (Figure 7). The downregulation of these genes was related to increased recognition memory. When taking diet into account, the presence of a relationship was highly variable. The relationship between the recognition index and *GRIN1* was driven by the control group, the relationship between the recognition index and *HDAC5* was driven by the control and BMOS + HMO group, and only an overall relationship was observed between the recognition index and *GABRR1* or *SLC1A7* (Figure 7).

How consumption of oligosaccharides affects these genes is unclear. 2′-Fucosyllactose has been shown to alter post-synaptic density protein 95, phosphorylated Ca^2+^/calmodulin-dependent protein kinase, and brain derived neurotrophic factor (Vázquez et al., 2015), but more evidence on the effect of prebiotics on gene expression in the brain has been generated from research on GOS. Brain derived neurotrophic factor, N-methyl-D-asparate receptor (NMDAR), and NMDAR subunits GluN1, GluN2A, and GluN2B in the hippocampus or cortex have been shown to be affected by prebiotic intake (Savignac et al., 2013, 2016; Williams et al., 2016; Gronier et al., 2018; Kao et al., 2018). In some cases, prebiotic intake was found not to impact structural/growth related proteins such as microtubule-associated-protein-2 and growth-associated-protein-43, perhaps suggesting a role of prebiotics in the modulation of molecular rather than structural components in the brain. We had previously hypothesized that HDACs may be involved in cognition via the inhibitory action of short-chain fatty acids, fermentative end products of microbial digestion in the colon. Short-chain fatty acids are known to act indirectly via transcriptional regulation as HDAC inhibitors or directly to modulate satiety (Frost et al., 2014), neurogenesis (Kim et al., 2009), and blood-brain-barrier integrity (Braniste et al., 2014). However, evidence that local signaling via the vagus nerve or the circulation and absorption of butyrate and/or acetate in the blood and brain is lacking. It is tempting to put forth the hypothesis that HDAC5 is a key regulator of cognitive development in context of OS consumption, however it is important to keep in mind that these two effects were simply co-occurring, and there may be an unmeasured variable that better explains the effect of oligosaccharide intake on cognition.

Initial evidence demonstrated that impairment of fucosylation in the rat hippocampus attenuates memory retention during a discrimination task (Jork et al., 1986). Conversely, incorporation of fucose into glycoproteins in chicks increased after a passive avoidance task (Sukumar et al., 1980). These studies demonstrated that fucosylation plays a large role in memory processes. Since then, studies have shown that administration of fucose or 2′-FL impacts long-term potentiation (LTP) in the hippocampus, a molecular process that facilitates synaptic transmission and is believed to be the mechanism of long-term memory storage in the hippocampus (Baudry, 2001). Rats intrahippocampally injected with L-fucose or 2′-FL demonstrated prolonged LTP duration compared to those injected with lactose (Krug et al., 1994). This effect was later replicated *ex vivo*, as bath application of L-fucose and 2′-FL, but not D-Fucose or 3′-FL, to rat hippocampal slices enhanced LTP by increasing the field excitatory postsynaptic potential and potentiation of the population spike amplitude (Matthies et al., 1996).

Although these studies demonstrated a strong link between fucose and memory function, the administration of fucose was independent of the diet, thus fucose and 2′-fucosyllactose bypassed the gastrointestinal system. More recent work demonstrated that rats and mice fed 2′-FL demonstrated increased and longer lasting potentiation of Schaffer collateral neurons in the CA1 region of the hippocampus (Vázquez et al., 2015). These animals also displayed increased performance on place learning, working memory, and fixed-ratio lever pressing tasks in an operant box, suggesting multiple cognitive domains were enhanced by 2′-FL intake. A follow up study from the same lab demonstrated that rats fed L-fucose did not demonstrate the same enhancement of LTP as those fed 2′-FL, and subdiaphragmatic bilateral vagotomy abolished the 2′-FL dependent increase in LTP (Vázquez et al., 2016). This study provided two critical components to understanding the mechanism of action behind 2′-FL mediated increases in cognition. First, ingestion of intact 2′-FL and not L-fucose is required for cognitive enhancement. Second, the cognitive enhancing effect of 2′-FL is mediated by the vagus nerve. Therefore, although 2′-FL is absorbed by the gut as evidenced by increased concentrations in plasma and urine in infants (Marriage et al., 2015), it is likely that absorbed 2′-FL does not act directly on the brain to promote cognition, but indirectly through vagal communication between the gut and brain. These data strongly suggest that although fucose is present as a glycoconjugate in the brain, the intake of fucose is not the primary driver of enhanced cognition.

If 2′-FL acts via some prebiotic action to improve cognition, this would be consistent with the observation that monosaccharide-based OS with prebiotic activity (e.g., FOS or GOS) are also capable of improving cognition (Kao et al., 2016). The hypothesis that all OS act via some specific prebiotic action to improve cognition provides a unifying explanation for the underlying mechanism but does not explain the differences observed between BMOS and HMO in the present study. If HMO are unique compared to other OS in the properties they impart to the immune system (Comstock et al., 2017), perhaps they might also be unique in their cognitive promoting abilities.

## Conclusion

To date, early life supplementation with oligosaccharides has generally been shown to either impart neutral or beneficial effects on cognition and brain development. Here, we investigated the impact of early life dietary supplementation with human and/or BMOS on cognition, structural brain development, and hippocampal mRNA expression. The brain regions affected included both cortical and subcortical regions yet measures of myelination or spectroscopy were largely unaffected by the diet. Although mRNA expression of several genes was affected, those related to behavior were most closely involved in GABAergic, glutamatergic, and histone deacetylation pathways. These relationships were highly dependent on the diet consumed, further highlighting the need to delineate the mechanisms behind gut-brain-axis communication and oligosaccharide consumption.

## Data Availability Statement

The datasets presented in this article are not readily available because they are the property of Nestlé. Requests to access the datasets should be directed to Ryan Dilger, rdilger2@illinois.edu.

## Ethics Statement

The animal study was reviewed and approved by University of Illinois at Urbana-Champaign Institutional Animal Care and Use Committee.

## Author Contributions

SF, AM, JY, JH, PS, SD, and RD contributed to the study design and interpretation. SF and AM contributed to the study execution. SM performed the gene expression analysis. SF, AM, and RD, performed the statistical analysis. All authors read and approved the final version of the manuscript.

## Conflict of Interest

JH, JY, SM, and PS are employees of Nestlé. SD and RD have consulted for and received grant funding from Nestlé. SF and RD both share ownership in Traverse Science. The remaining author declares that the research was conducted in the absence of any commercial or financial relationships that could be construed as a potential conflict of interest.

## Footnotes

1 http://pigmri.illinois.edu
