# Supplementary material for: Human and Bovine Milk Oligosaccharides Elicit Improved Recognition Memory Concurrent With Alterations in Regional Brain Volumes and Hippocampal mRNA Expression
Source: Front Neurosci. 2020 Aug 13;14:770. doi: 10.3389/fnins.2020.00770 (PMC7438728; doi:10.3389/fnins.2020.00770)
Supplement: Supplementary file 1 [file Table_1.DOCX]

Human and bovine milk oligosaccharides elicit improved recognition memory concurrent with alterations in regional brain volumes and hippocampal mRNA expression

Stephen A. Fleming^1,2^, Austin T. Mudd^1,2^, Jonas Hauser^3^, Jian Yan^4^, Sylviane Metairon^3^, Pascal Steiner^3^, Sharon M. Donovan^5,6^, and Ryan N. Dilger^1,2,5,6*^

^1^Piglet Nutrition and Cognition Laboratory, Department of Animal Sciences, University of Illinois, Urbana, IL, USA

^2^Neuroscience Program, University of Illinois, Urbana, Illinois, USA

^3^Nestlé Research, Lausanne, Switzerland

^4^Nestlé Product Technology Center Nutrition, Vevey, Switzerland

^5^Department of Food Science and Human Nutrition, University of Illinois, Urbana, IL, USA

^6^Division of Nutritional Sciences, University of Illinois, Urbana, Illinois, USA

*Correspondence:

Dr. Ryan N. Dilger

University of Illinois

Department of Animal Sciences

Piglet Nutrition and Cognition Laboratory

1207 W. Gregory Street

186 Animal Sciences Laboratory

Urbana, IL, 61801, USA

(217) 333-2006

[rdilger2@illinois.edu](mailto:rdilger2@illinois.edu)

| **Supplemental Table 1**. Exploratory behavior during the novel object recognition task^1,2^ | | | | | | | | | | | | |
| --- | --- | --- | --- | --- | --- | --- | --- | --- | --- | --- | --- | --- |
|  | **Diet** | | | | | | | |  |  | | |
|  | **CON** | | **BMOS** | | **HMO** | | **BMOS + HMO** | | **Pooled** | **P-value^3^** | | |
| **Measure** | **N** | **Mean** | **N** | **Mean** | **N** | **Mean** | **N** | **Mean** | **SEM** | **BMOS** | **HMO** | **INT** |
| Habituation 1 |  |  |  |  |  |  |  |  |  |  |  |  |
| Total distance moved, m | 12 | 119^a^ | 12 | 103^ab^ | 12 | 85^b^ | 11 | 109^ab^ | 7 | 0.655 | 0.057 | 0.008 |
| Δ distance moved, m/min | 12 | 2.0 | 12 | 1.0 | 12 | 1.4 | 11 | 0.5 | 0.4 | 0.027 | 0.163 | 0.912 |
| Time spent in the center of the arena, % | 12 | 67 | 12 | 68 | 12 | 66 | 11 | 59 | 3 | 0.304 | 0.133 | 0.228 |
| Habituation 2 |  |  |  |  |  |  |  |  |  |  |  |  |
| Total distance moved, m | 12 | 81 | 12 | 63 | 12 | 75 | 11 | 74 | 9 | 0.277 | 0.812 | 0.350 |
| Δ distance moved, m/min | 12 | 0.3 | 12 | 0.2 | 12 | 0.6 | 11 | 0.5 | 0.3 | 0.622 | 0.223 | 0.953 |
| Time spent in the center of the arena, % | 12 | 58 | 12 | 55 | 12 | 63 | 11 | 56 | 5 | 0.330 | 0.558 | 0.663 |
| Sample Phase |  |  |  |  |  |  |  |  |  |  |  |  |
| Total object visit time, s | 12 | 77.5 | 12 | 78.5 | 12 | 111.5 | 11 | 105.4 | 15.6 | 0.854 | 0.032 | 0.798 |
| Number of object visits | 12 | 11.9 | 12 | 11.5 | 12 | 12.7 | 11 | 13.2 | 1.5 | 0.954 | 0.381 | 0.763 |
| Mean object visit time, s | 12 | 6.7 | 11 | 5.9 | 12 | 8.9 | 11 | 8.3 | 1.3 | 0.543 | 0.053 | 0.945 |
| Latency to first object visit, s | 11 | 21.3 | 11 | 14.4 | 12 | 10.4 | 11 | 13.9 | 6.1 | 0.778 | 0.348 | 0.392 |
| Habituation towards both objects, s/min | 12 | -3.4 | 12 | -2.8 | 12 | -5.3 | 11 | -4.2 | 1.3 | 0.466 | 0.147 | 0.860 |
| Total distance moved, m | 12 | 32 | 12 | 30 | 12 | 28 | 11 | 32 | 3 | 0.670 | 0.750 | 0.338 |
| Δ distance moved, m/min | 12 | 0.4 | 12 | 0.3 | 12 | 0.2 | 11 | 0.2 | 0.2 | 0.899 | 0.314 | 0.780 |
| Time spent in the center of the arena, % | 12 | 42 | 12 | 46 | 12 | 41 | 11 | 36 | 5 | 0.973 | 0.254 | 0.355 |
| ^1^Abbreviations: CON, control group; BMOS, pigs fed bovine milk oligosaccharides; HMO, pigs fed 2’fucosyllactose and Lacto-N-neotetraose; SEM, standard error of the mean; Int, interaction effect of BMOS and HMO; m, meter; Δ, change in; min, minute; %, percent; s, second. | | | | | | | | | | | | |
| ^2^Data presented are least square means. | | | | | | | | | | | | |
| *^3^P*-values derived from mixed model ANOVA. | | | | | | | | | | | | |

| **Supplemental Table 1**. Continued^1,2^ | | | | | | | | | | | | |
| --- | --- | --- | --- | --- | --- | --- | --- | --- | --- | --- | --- | --- |
|  | **Diet** | | | | | | | |  |  | | |
|  | **CON** | | **BMOS** | | **HMO** | | **BMOS + HMO** | | **Pooled** | **P-value^3^** | | |
| **Measure** | **N** | **Mean** | **N** | **Mean** | **N** | **Mean** | **N** | **Mean** | **SEM** | **BMOS** | **HMO** | **INT** |
| Test Phase: 1-hour delay |  |  |  |  |  |  |  |  |  |  |  |  |
| Novel object visit time, s | 9 | 43.1 | 11 | 40.7 | 11 | 60.0 | 11 | 58.9 | 13.5 | 0.877 | 0.126 | 0.954 |
| Number of novel object visits | 11 | 4.3 | 12 | 5.5 | 12 | 4.7 | 11 | 5.8 | 0.9 | 0.122 | 0.638 | 0.985 |
| Mean novel object visit time, s | 11 | 9.6 | 12 | 6.7 | 12 | 12.5 | 11 | 9.5 | 1.9 | 0.110 | 0.117 | 0.967 |
| Latency to first novel object visit, s | 9 | 29.3 | 11 | 22.4 | 11 | 30.8 | 11 | 14.0 | 10.3 | 0.136 | 0.659 | 0.521 |
| Habituation to the novel object, s/min | 10 | -1.5 | 11 | -2.7 | 11 | -2.6 | 11 | -3.6 | 1.5 | 0.455 | 0.463 | 0.934 |
| Sample object visit time, s | 10 | 38.1 | 10 | 38.9 | 12 | 42.9 | 10 | 36.9 | 11.1 | 0.775 | 0.876 | 0.705 |
| Number of sample object visits | 11 | 5.6 | 12 | 5.1 | 12 | 5.3 | 11 | 5.9 | 1.0 | 0.930 | 0.636 | 0.420 |
| Mean sample object visit time, s | 11 | 6.3 | 12 | 7.8 | 12 | 8.2 | 11 | 5.0 | 1.4 | 0.462 | 0.741 | 0.053 |
| Latency to first sample object visit, s | 9 | 10.3 | 10 | 25.4 | 11 | 17.9 | 11 | 29.9 | 7.3 | 0.056 | 0.381 | 0.821 |
| Habituation to the sample object, s/min | 10 | -2.1 | 10 | -1.0 | 12 | -0.7 | 11 | 0.0 | 1.3 | 0.449 | 0.321 | 0.872 |
| Total object visit time, s | 11 | 81.0 | 12 | 79.6 | 12 | 102.9 | 11 | 95.6 | 20.2 | 0.787 | 0.241 | 0.853 |
| Number of object visits | 11 | 9.0 | 12 | 10.6 | 12 | 10.0 | 11 | 10.8 | 1.8 | 0.372 | 0.654 | 0.749 |
| Mean object visit time, s | 11 | 8.4 | 11 | 7.0 | 12 | 9.8 | 11 | 8.0 | 1.4 | 0.248 | 0.370 | 0.878 |
| Latency to first object visit, s | 10 | 8.1 | 11 | 15.9 | 12 | 18.4 | 11 | 12.0 | 5.5 | 0.872 | 0.473 | 0.115 |
| Habituation towards both objects, s/min | 11 | -3.4 | 11 | -3.6 | 12 | -3.1 | 11 | -3.6 | 1.9 | 0.851 | 0.946 | 0.938 |
| Total distance moved, m | 11 | 29 | 12 | 28 | 12 | 27 | 11 | 30 | 3 | 0.856 | 0.922 | 0.439 |
| Δ distance moved, m/min | 11 | 0.2 | 12 | -0.1 | 11 | 0.3 | 11 | -0.1 | 0.2 | 0.149 | 0.943 | 0.660 |
| Time spent in the center of the arena, % | 11 | 39 | 12 | 37 | 12 | 41 | 11 | 34 | 5 | 0.427 | 0.914 | 0.654 |
| ^1^Abbreviations: CON, control group; BMOS, pigs fed bovine milk oligosaccharides; HMO, pigs fed 2’fucosyllactose and Lacto-N-neotetraose; SEM, standard error of the mean; Int, interaction effect of BMOS and HMO; m, meter; min, minute; %, percent; s, second; Δ, change in. | | | | | | | | | | | | |
| ^2^Data presented are least square means. | | | | | | | | | | | | |
| *^3^P*-values derived from mixed model ANOVA. | | | | | | | | | | | | |

| **Supplemental Table 1**. Continued^1,2^ | | | | | | | | | | | | |
| --- | --- | --- | --- | --- | --- | --- | --- | --- | --- | --- | --- | --- |
|  | **Diet** | | | | | | | |  |  | | |
|  |  | **CON** |  | **BMOS** |  | **HMO** |  | **BMOS + HMO** | **Pooled** | **P-value^3^** | | |
| **Measure** | **N** | **Mean** | **N** | **Mean** | **N** | **Mean** | **N** | **Mean** | **SEM** | **BMOS** | **HMO** | **INT** |
| Test Phase: 48-hour delay |  |  |  |  |  |  |  |  |  |  |  |  |
| Novel object visit time, s | 10 | 37.2 | 10 | 44.9 | 11 | 36.7 | 11 | 49.5 | 11.8 | 0.381 | 0.855 | 0.827 |
| Number of novel object visits | 11 | 5.2 | 12 | 4.3 | 12 | 4.6 | 11 | 6.3 | 0.9 | 0.639 | 0.456 | 0.162 |
| Mean novel object visit time, s | 11 | 7.3 | 12 | 7.0 | 12 | 7.3 | 11 | 6.6 | 1.6 | 0.722 | 0.905 | 0.912 |
| Latency to first novel object visit, s | 10 | 23.2 | 11 | 50.4 | 10 | 17.6 | 11 | 26.6 | 14.2 | 0.179 | 0.272 | 0.492 |
| Habituation to the novel object, s/min | 10 | -2.3 | 11 | -4.4 | 11 | -1.2 | 11 | -1.4 | 1.4 | 0.305 | 0.065 | 0.389 |
| Sample object visit time, s | 11 | 33.5 | 11 | 30.3 | 11 | 32.4 | 11 | 32.8 | 8.2 | 0.864 | 0.928 | 0.828 |
| Number of sample object visits | 11 | 5.5 | 12 | 4.8 | 12 | 4.5 | 11 | 6.0 | 1.0 | 0.644 | 0.911 | 0.268 |
| Mean sample object visit time, s | 11 | 5.9 | 11 | 8.8 | 12 | 5.5 | 11 | 5.6 | 1.8 | 0.384 | 0.304 | 0.422 |
| Latency to first sample object visit, s | 11 | 42.4 | 10 | 15.6 | 11 | 28.8 | 11 | 29.2 | 10.3 | 0.191 | 0.999 | 0.178 |
| Habituation to the sample object, s/min | 10 | 0.3 | 10 | -0.2 | 12 | 0.1 | 11 | -1.2 | 1.0 | 0.309 | 0.506 | 0.689 |
| Total object visit time, s | 11 | 70.6 | 12 | 84.6 | 12 | 69.2 | 11 | 82.3 | 17.7 | 0.440 | 0.915 | 0.981 |
| Number of object visits | 11 | 9.8 | 12 | 9.2 | 12 | 9.1 | 11 | 11.3 | 1.7 | 0.645 | 0.680 | 0.425 |
| Mean object visit time, s | 11 | 6.3 | 10 | 6.9 | 12 | 7.2 | 11 | 6.6 | 1.5 | 0.994 | 0.818 | 0.654 |
| Latency to first object visit, s | 10 | 9.1 | 12 | 9.6 | 11 | 7.8 | 11 | 18.4 | 4.0 | 0.164 | 0.349 | 0.204 |
| Habituation towards both objects, s/min | 10 | -2.2 | 11 | -3.0 | 12 | -1.0 | 11 | -2.5 | 1.3 | 0.363 | 0.492 | 0.769 |
| Total distance moved, m | 11 | 33 | 12 | 29 | 12 | 27 | 11 | 33 | 3 | 0.833 | 0.728 | 0.119 |
| Δ distance moved, m/min | 11 | -0.3 | 11 | -0.3 | 12 | -0.4 | 11 | -0.3 | 0.2 | 0.907 | 0.904 | 0.986 |
| Time spent in the center of the arena, % | 11 | 38 | 12 | 42 | 12 | 50 | 11 | 39 | 6 | 0.526 | 0.470 | 0.248 |
| ^1^Abbreviations: CON, control group; BMOS, pigs fed bovine milk oligosaccharides; HMO, pigs fed 2’fucosyllactose and Lacto-N-neotetraose; SEM, standard error of the mean; Int, interaction effect of BMOS and HMO; m, meter; min, minute; %, percent; s, second; Δ, change in. | | | | | | | | | | | | |
| ^2^Data presented are least square means. | | | | | | | | | | | | |
| *^3^P*-values derived from mixed model ANOVA. | | | | | | | | | | | | |

| **Supplemental Table 2.** Absolute Brain Volume^1,2^ | | | | | | | | | | | | |
| --- | --- | --- | --- | --- | --- | --- | --- | --- | --- | --- | --- | --- |
|  | **Diet** | | | | | | | |  |  | | |
|  | **CON** | | **BMOS** | | **HMO** | | **BMOS + HMO** | | **Pooled** | ***P*-Value^3^** | | |
| **Brain Region, mm^3^** | **N** | **Mean** | **N** | **Mean** | **N** | **Mean** | **N** | **Mean** | **SEM** | **BMOS** | **HMO** | **INT** |
| Caudate^4^ | 11 | 502.1^a^ | 12 | 480.7^a^ | 10 | 457.5^a^ | 9 | 485.9^a^ | 13.69 | 0.770 | 0.113 | 0.047 |
| Cerebellum | 11 | 6108.4 | 12 | 5899.6 | 10 | 5938.3 | 9 | 6050.9 | 127.34 | 0.678 | 0.935 | 0.172 |
| Cerebral Aqueduct | 11 | 47.1 | 12 | 44.1 | 10 | 45.6 | 9 | 45.7 | 1.81 | 0.394 | 0.991 | 0.352 |
| Corpus Callosum | 11 | 374.3 | 11 | 354.2 | 10 | 367.5 | 9 | 366.4 | 9.10 | 0.107 | 0.681 | 0.150 |
| Cerebrospinal Fluid | 11 | 5302.9 | 12 | 6203.0 | 10 | 6750.4 | 9 | 5916.7 | 593.96 | 0.952 | 0.301 | 0.126 |
| Fourth Ventricle | 11 | 51.7 | 12 | 50.4 | 10 | 50.3 | 9 | 52.4 | 2.93 | 0.869 | 0.907 | 0.544 |
| Grey Matter^4^ | 11 | 37864.2^a^ | 12 | 36000.9^a^ | 10 | 35348.4^a^ | 9 | 37453.8^a^ | 983.50 | 0.877 | 0.499 | 0.016 |
| Hypothalamus^4^ | 11 | 249.7^a^ | 11 | 232.0^a^ | 9 | 230.6^a^ | 9 | 245.8^a^ | 5.54 | 0.813 | 0.621 | 0.004 |
| Internal Capsule | 11 | 1535.6 | 12 | 1496.7 | 10 | 1494.7 | 9 | 1538.7 | 37.35 | 0.932 | 0.985 | 0.171 |
| Lateral Ventricle | 11 | 492.0 | 12 | 485.2 | 10 | 522.0 | 8 | 464.3 | 17.56 | 0.047 | 0.775 | 0.114 |
| Left Cortex | 11 | 16709.3 | 12 | 16350.0 | 10 | 16018.9 | 9 | 16757.7 | 356.16 | 0.532 | 0.642 | 0.078 |
| Left Hippocampus | 11 | 480.6 | 12 | 463.9 | 10 | 460.6 | 9 | 474.2 | 12.50 | 0.876 | 0.623 | 0.128 |
| Medulla | 11 | 1835.1 | 11 | 1837.9 | 10 | 1817.7 | 9 | 1824.2 | 35.83 | 0.875 | 0.605 | 0.950 |
| Midbrain | 11 | 1874.4 | 12 | 1856.2 | 10 | 1860.1 | 9 | 1862.1 | 33.45 | 0.797 | 0.895 | 0.748 |
| Olfactory Bulb | 11 | 2319.6 | 11 | 2363.4 | 10 | 2374.8 | 9 | 2337.5 | 59.51 | 0.947 | 0.765 | 0.411 |
| Pons | 11 | 1082.1 | 12 | 1094.9 | 10 | 1084.8 | 9 | 1068.9 | 25.28 | 0.944 | 0.595 | 0.512 |
| Putamen-Globus Pallidus | 11 | 414.8 | 12 | 400.6 | 10 | 396.9 | 9 | 409.3 | 9.00 | 0.909 | 0.580 | 0.114 |
| Right Cortex | 11 | 17242.8 | 12 | 16772.4 | 10 | 16615.3 | 9 | 17233.5 | 409.88 | 0.816 | 0.795 | 0.096 |
| Right Hippocampus | 11 | 490.6 | 12 | 477.6 | 10 | 471.7 | 9 | 492.3 | 12.38 | 0.704 | 0.836 | 0.100 |
| Thalamus^4^ | 11 | 1638.4^a^ | 12 | 1553.4^a^ | 10 | 1535.7^a^ | 9 | 1622.3^a^ | 30.71 | 0.978 | 0.545 | 0.004 |
| Third Ventricle | 11 | 48.9 | 12 | 48.7 | 10 | 45.9 | 9 | 48.3 | 2.05 | 0.560 | 0.382 | 0.488 |
| White Matter | 11 | 16938.1 | 12 | 16477.9 | 10 | 16881.1 | 9 | 16928.3 | 541.43 | 0.627 | 0.645 | 0.554 |
| Whole Brain | 11 | 66375.0 | 12 | 65028.9 | 10 | 63549.8 | 9 | 65214.1 | 1556.45 | 0.904 | 0.321 | 0.259 |
| ^1^Abbreviations: mm, millimeter; CON, control group; BMOS, pigs fed bovine milk oligosaccharides; HMO, pigs fed 2’fucosyllactose and Lacto-N-neotetraose; SEM, standard error of the mean; Int, interaction effect of BMOS and HMO. | | | | | | | | | | | | |
| ^2^Data presented are least square means. | | | | | | | | | | | | |
| *^3^P*-values derived from mixed model ANOVA. | | | | | | | | | | | | |
| ^4^Mean separation insignificant after Tukey adjustment. | | | | | | | | | | | | |
| ^abc^Means without a common superscript differ (P < 0.05). | | | | | | | | | | | | |

| **Supplemental Table 3**. Relative Brain Volume^1,2^ | | | | | | | | | | | | |
| --- | --- | --- | --- | --- | --- | --- | --- | --- | --- | --- | --- | --- |
|  | **Diet** | | | | | | | |  |  | | |
|  | **CON** | | **BMOS** | | **HMO** | | **BMOS + HMO** | | **Pooled** | ***P*-Value^3^** | | |
| **Brain Region, %TBV** | **N** | **Mean** | **N** | **Mean** | **N** | **Mean** | **N** | **Mean** | **SEM** | **BMOS** | **HMO** | **INT** |
| Caudate^4^ | 11 | 0.758^a^ | 11 | 0.728^a^ | 10 | 0.720^a^ | 9 | 0.747^a^ | 0.016 | 0.924 | 0.422 | 0.020 |
| Cerebellum | 11 | 9.211 | 12 | 9.100 | 10 | 9.360 | 9 | 9.269 | 0.172 | 0.457 | 0.247 | 0.943 |
| Cerebral Aqueduct | 11 | 0.071 | 12 | 0.068 | 10 | 0.072 | 9 | 0.070 | 0.003 | 0.401 | 0.618 | 0.902 |
| Corpus Callosum | 11 | 0.567 | 11 | 0.547 | 10 | 0.580 | 9 | 0.567 | 0.016 | 0.038 | 0.042 | 0.677 |
| Cerebrospinal Fluid | 11 | 7.930 | 12 | 9.604 | 10 | 10.676 | 9 | 9.040 | 0.903 | 0.982 | 0.198 | 0.054 |
| Fourth Ventricle | 11 | 0.077 | 12 | 0.078 | 10 | 0.079 | 9 | 0.080 | 0.004 | 0.939 | 0.639 | 0.972 |
| Grey Matter | 11 | 57.213 | 11 | 56.391 | 10 | 55.718 | 9 | 57.741 | 0.755 | 0.404 | 0.919 | 0.054 |
| Hypothalamus | 11 | 0.376 | 11 | 0.358 | 10 | 0.376 | 9 | 0.376 | 0.009 | 0.265 | 0.271 | 0.312 |
| Internal Capsule | 11 | 2.325 | 12 | 2.309 | 10 | 2.363 | 9 | 2.378 | 0.064 | 0.990 | 0.144 | 0.664 |
| Lateral Ventricle | 11 | 0.749^ab^ | 12 | 0.750^ab^ | 10 | 0.832^a^ | 8 | 0.730^b^ | 0.036 | 0.033 | 0.174 | 0.034 |
| Left Cortex | 11 | 25.194 | 12 | 25.172 | 9 | 25.503 | 9 | 25.675 | 0.245 | 0.663 | 0.023 | 0.576 |
| Left Hippocampus | 11 | 0.725 | 12 | 0.714 | 10 | 0.725 | 9 | 0.730 | 0.013 | 0.747 | 0.402 | 0.408 |
| Medulla | 11 | 2.791 | 12 | 2.790 | 10 | 2.881 | 9 | 2.846 | 0.093 | 0.678 | 0.099 | 0.693 |
| Midbrain | 11 | 2.830 | 12 | 2.865 | 10 | 2.932 | 9 | 2.865 | 0.055 | 0.707 | 0.241 | 0.239 |
| Olfactory Bulb | 11 | 3.499 | 11 | 3.645 | 10 | 3.741 | 9 | 3.586 | 0.082 | 0.953 | 0.231 | 0.054 |
| Pons^4^ | 11 | 1.629^a^ | 12 | 1.690^a^ | 10 | 1.709^a^ | 9 | 1.640^a^ | 0.030 | 0.856 | 0.549 | 0.014 |
| Putamen-Globus Pallidus | 11 | 0.627 | 12 | 0.618 | 10 | 0.625 | 9 | 0.630 | 0.014 | 0.794 | 0.590 | 0.479 |
| Right Cortex | 11 | 26.027 | 12 | 25.802 | 9 | 26.449 | 9 | 26.477 | 0.243 | 0.595 | 0.005 | 0.498 |
| Right Hippocampus | 11 | 0.740 | 12 | 0.735 | 10 | 0.742 | 9 | 0.756 | 0.011 | 0.552 | 0.144 | 0.260 |
| Thalamus | 11 | 2.469 | 12 | 2.398 | 10 | 2.419 | 9 | 2.481 | 0.042 | 0.901 | 0.624 | 0.058 |
| Third Ventricle | 11 | 0.073 | 12 | 0.075 | 10 | 0.072 | 9 | 0.074 | 0.003 | 0.582 | 0.689 | 0.932 |
| White Matter | 11 | 25.673 | 12 | 25.422 | 9 | 26.059 | 9 | 26.225 | 0.831 | 0.926 | 0.195 | 0.649 |
| ^1^Abbreviations: %TBV, percent total brain volume; CON, control group; BMOS, pigs fed bovine milk oligosaccharides; HMO, pigs fed 2’fucosyllactose and Lacto-N-neotetraose; SEM, standard error of the mean; Int, interaction effect of BMOS and HMO. | | | | | | | | | | | | |
| ^2^Data presented are least square means. | | | | | | | | | | | | |
| *^3^P*-values derived from mixed model ANOVA. | | | | | | | | | | | | |
| ^4^Mean separation insignificant after Tukey adjustment. | | | | | | | | | | | | |
| ^abc^Means without a common superscript differ (P < 0.05). | | | | | | | | | | | | |

| **Supplemental Table 4**. Diffusion Tensor Imaging^1,2^ | | | | | | | | | | | | |
| --- | --- | --- | --- | --- | --- | --- | --- | --- | --- | --- | --- | --- |
|  | **Diet** | | | | | | | |  |  | | |
|  | **CON** | | **BMOS** | | **HMO** | | **BMOS + HMO** | | **Pooled** | ***P*-Value** | | |
| **Measure** | **N** | **Mean** | **N** | **Mean** | **N** | **Mean** | **N** | **Mean** | **SEM** | **BMOS** | **HMO** | **INT** |
| Axial diffusivity, x10^-3^ mm^2^/s |  |  |  |  |  |  |  |  |  |  |  |  |
| Caudate | 11 | 1.188 | 12 | 1.222 | 10 | 1.221 | 9 | 1.227 | 0.023 | 0.361 | 0.363 | 0.520 |
| Corpus Callosum | 11 | 1.616 | 12 | 1.668 | 10 | 1.649 | 9 | 1.682 | 0.060 | 0.397 | 0.639 | 0.844 |
| Cerebellum | 11 | 1.347 | 12 | 1.427 | 10 | 1.329 | 9 | 1.351 | 0.036 | 0.143 | 0.173 | 0.401 |
| Left Hippocampus | 10 | 1.516 | 12 | 1.531 | 10 | 1.496 | 9 | 1.492 | 0.039 | 0.878 | 0.427 | 0.801 |
| Right Hippocampus | 11 | 1.504 | 12 | 1.510 | 10 | 1.489 | 9 | 1.489 | 0.047 | 0.931 | 0.640 | 0.941 |
| Internal Capsule | 11 | 1.277 | 12 | 1.286 | 10 | 1.269 | 9 | 1.286 | 0.009 | 0.112 | 0.621 | 0.626 |
| Left Cortex | 11 | 1.363 | 12 | 1.355 | 10 | 1.358 | 9 | 1.358 | 0.011 | 0.649 | 0.874 | 0.647 |
| Right Cortex | 11 | 1.401 | 12 | 1.419 | 10 | 1.436 | 9 | 1.413 | 0.023 | 0.903 | 0.432 | 0.258 |
| Thalamus | 10 | 1.180 | 12 | 1.165 | 10 | 1.162 | 9 | 1.171 | 0.016 | 0.836 | 0.700 | 0.401 |
| DTI-derived white matter | 11 | 1.406 | 12 | 1.401 | 10 | 1.401 | 9 | 1.399 | 0.013 | 0.743 | 0.760 | 0.921 |
| Atlas-derived white matter | 11 | 1.457 | 12 | 1.460 | 10 | 1.450 | 9 | 1.451 | 0.017 | 0.874 | 0.464 | 0.899 |
| Radial diffusivity, x10^-3^ mm^2^/s |  |  |  |  |  |  |  |  |  |  |  |  |
| Caudate | 11 | 0.706 | 11 | 0.717 | 10 | 0.746 | 9 | 0.727 | 0.014 | 0.782 | 0.063 | 0.250 |
| Corpus Callosum | 11 | 1.023 | 12 | 1.059 | 10 | 1.054 | 9 | 1.065 | 0.039 | 0.441 | 0.544 | 0.680 |
| Cerebellum | 11 | 1.054 | 12 | 1.121 | 10 | 1.037 | 9 | 1.044 | 0.032 | 0.224 | 0.129 | 0.328 |
| Left Hippocampus | 10 | 0.940 | 12 | 0.966 | 10 | 0.935 | 9 | 0.931 | 0.029 | 0.684 | 0.455 | 0.570 |
| Right Hippocampus | 11 | 0.943 | 12 | 0.964 | 10 | 0.933 | 9 | 0.933 | 0.033 | 0.704 | 0.467 | 0.705 |
| Internal Capsule | 10 | 0.660 | 12 | 0.653 | 10 | 0.666 | 9 | 0.655 | 0.007 | 0.196 | 0.560 | 0.717 |
| Left Cortex | 11 | 0.849 | 12 | 0.839 | 10 | 0.842 | 9 | 0.837 | 0.008 | 0.294 | 0.570 | 0.740 |
| Right Cortex | 11 | 0.860 | 12 | 0.871 | 10 | 0.885 | 9 | 0.858 | 0.017 | 0.580 | 0.676 | 0.183 |
| Thalamus | 11 | 0.720 | 12 | 0.730 | 10 | 0.723 | 9 | 0.726 | 0.008 | 0.379 | 0.889 | 0.625 |
| DTI-derived white matter | 11 | 0.878 | 12 | 0.871 | 10 | 0.873 | 9 | 0.866 | 0.010 | 0.382 | 0.537 | 0.966 |
| Atlas-derived white matter | 11 | 0.910 | 11 | 0.903 | 10 | 0.906 | 9 | 0.900 | 0.013 | 0.380 | 0.574 | 0.989 |
| ^1^Abbreviations: mm, millimeter; s, second; CON, control group; BMOS, pigs fed bovine milk oligosaccharides; HMO, pigs fed 2’fucosyllactose and Lacto-N-neotetraose; SEM, standard error of the mean; Int, interaction effect of BMOS and HMO; DTI, diffusion tensor imaging. | | | | | | | | | | | | |
| ^2^Data presented are least square means. | | | | | | | | | | | | |
| *^3^P*-values derived from mixed model ANOVA. | | | | | | | | | | | | |

| **Supplemental Table 4**. Continued^1,2^ | | | | | | | | | | | | |
| --- | --- | --- | --- | --- | --- | --- | --- | --- | --- | --- | --- | --- |
|  | **Diet** | | | | | | | |  |  | | |
|  | **CON** | | **BMOS** | | **HMO** | | **BMOS + HMO** | | **Pooled** | ***P*-Value** | | |
| **Measure** | **N** | **Mean** | **N** | **Mean** | **N** | **Mean** | **N** | **Mean** | **SEM** | **BMOS** | **HMO** | **INT** |
| Mean diffusivity, x10^-3^ mm^2^/s |  |  |  |  |  |  |  |  |  |  |  |  |
| Caudate | 11 | 0.867 | 11 | 0.882 | 10 | 0.904 | 9 | 0.894 | 0.016 | 0.875 | 0.102 | 0.391 |
| Corpus Callosum | 11 | 1.221 | 12 | 1.262 | 10 | 1.253 | 9 | 1.271 | 0.045 | 0.417 | 0.580 | 0.753 |
| Cerebellum | 11 | 1.152 | 12 | 1.223 | 10 | 1.134 | 9 | 1.147 | 0.033 | 0.190 | 0.142 | 0.351 |
| Left Hippocampus | 10 | 1.132 | 12 | 1.155 | 10 | 1.122 | 9 | 1.118 | 0.032 | 0.757 | 0.437 | 0.655 |
| Right Hippocampus | 11 | 1.130 | 12 | 1.146 | 10 | 1.118 | 9 | 1.119 | 0.037 | 0.794 | 0.531 | 0.799 |
| Internal Capsule | 10 | 0.866 | 12 | 0.864 | 10 | 0.867 | 9 | 0.865 | 0.006 | 0.743 | 0.887 | 0.971 |
| Left Cortex | 11 | 1.020 | 12 | 1.011 | 10 | 1.014 | 9 | 1.011 | 0.009 | 0.399 | 0.675 | 0.701 |
| Right Cortex | 11 | 1.040 | 12 | 1.054 | 10 | 1.069 | 9 | 1.043 | 0.019 | 0.695 | 0.569 | 0.202 |
| Thalamus | 11 | 0.868 | 11 | 0.866 | 10 | 0.869 | 9 | 0.873 | 0.009 | 0.885 | 0.567 | 0.715 |
| DTI-derived white matter | 11 | 1.054 | 12 | 1.048 | 10 | 1.049 | 9 | 1.044 | 0.011 | 0.502 | 0.616 | 0.991 |
| Atlas-derived white matter | 11 | 1.092 | 12 | 1.094 | 10 | 1.087 | 9 | 1.083 | 0.014 | 0.908 | 0.373 | 0.744 |
| Fractional Anisotropy, arbitrary units |  |  |  |  |  |  |  |  |  |  |  |  |
| Caudate | 11 | 0.333 | 11 | 0.333 | 10 | 0.318 | 9 | 0.336 | 0.006 | 0.106 | 0.278 | 0.102 |
| Corpus Callosum | 11 | 0.296 | 12 | 0.293 | 10 | 0.290 | 9 | 0.300 | 0.004 | 0.403 | 0.920 | 0.159 |
| Cerebellum | 10 | 0.167 | 12 | 0.168 | 10 | 0.165 | 9 | 0.175 | 0.004 | 0.243 | 0.582 | 0.318 |
| Left Hippocampus | 9 | 0.301 | 12 | 0.293 | 10 | 0.301 | 9 | 0.305 | 0.006 | 0.662 | 0.342 | 0.296 |
| Right Hippocampus | 10 | 0.291 | 12 | 0.285 | 10 | 0.293 | 9 | 0.291 | 0.005 | 0.323 | 0.317 | 0.648 |
| Internal Capsule | 10 | 0.409 | 12 | 0.416 | 10 | 0.401 | 9 | 0.414 | 0.006 | 0.069 | 0.389 | 0.605 |
| Left Cortex | 10 | 0.315 | 12 | 0.316 | 10 | 0.314 | 9 | 0.316 | 0.002 | 0.347 | 0.989 | 0.707 |
| Right Cortex | 10 | 0.329 | 12 | 0.325 | 10 | 0.322 | 9 | 0.327 | 0.002 | 0.800 | 0.273 | 0.064 |
| Thalamus | 11 | 0.313 | 12 | 0.306 | 10 | 0.309 | 9 | 0.311 | 0.008 | 0.682 | 0.936 | 0.558 |
| DTI-derived white matter | 10 | 0.315 | 12 | 0.314 | 10 | 0.313 | 9 | 0.316 | 0.002 | 0.425 | 0.940 | 0.350 |
| Atlas-derived white matter | 11 | 0.314 | 12 | 0.315 | 10 | 0.314 | 9 | 0.317 | 0.002 | 0.198 | 0.520 | 0.514 |
| ^1^Abbreviations: mm, millimeter; s, second; CON, control group; BMOS, pigs fed bovine milk oligosaccharides; HMO, pigs fed 2’fucosyllactose and Lacto-N-neotetraose; SEM, standard error of the mean; Int, interaction effect of BMOS and HMO; DTI, diffusion tensor imaging. | | | | | | | | | | | | |
| ^2^Data presented are least square means. | | | | | | | | | | | | |
| *^3^P*-values derived from mixed model ANOVA. | | | | | | | | | | | | |

| **Supplemental Table 5**. Magnetic Resonance Spectroscopy^1,2^ | | | | | | | | | | | | |
| --- | --- | --- | --- | --- | --- | --- | --- | --- | --- | --- | --- | --- |
|  | **Diet** | | | | | | | |  |  | | |
|  | **CON** | | **BMOS** | | **HMO** | | **BMOS + HMO** | | **Pooled** | ***P*-Value^3^** | | |
| **Metabolite, ppm** | **N** | **Mean** | **N** | **Mean** | **N** | **Mean** | **N** | **Mean** | **SEM** | **BMOS** | **HMO** | **INT** |
| Glutathione | 9 | 1.11 | 7 | 1.35 | 10 | 1.23 | 9 | 1.08 | 0.164 | 0.649 | 0.473 | 0.057 |
| Myo-inositol | 10 | 4.19 | 8 | 4.29 | 10 | 4.01 | 9 | 4.75 | 0.384 | 0.257 | 0.708 | 0.387 |
| N-acetylaspartate | 10 | 4.25 | 8 | 4.41 | 10 | 4.40 | 9 | 4.24 | 0.202 | 0.998 | 0.953 | 0.424 |
| γ-amino butyric acid | 11 | 1.39 | 8 | 1.39 | 11 | 1.42 | 9 | 1.45 | 0.063 | 0.810 | 0.443 | 0.797 |
| ^1^Abbreviations: ppm, parts per million; CON, control group; BMOS, pigs fed bovine milk oligosaccharides; HMO, pigs fed 2’fucosyllactose and Lacto-N-neotetraose; SEM, standard error of the mean; Int, interaction effect of BMOS and HMO. | | | | | | | | | | | | |
| ^2^Data presented are least square means. | | | | | | | | | | | | |
| *^3^P*-values derived from mixed model ANOVA. | | | | | | | | | | | | |

| **Supplemental Table 6**. Standardized mRNA expression^1^ | | | | | | | | | | | | | |
| --- | --- | --- | --- | --- | --- | --- | --- | --- | --- | --- | --- | --- | --- |
|  | **Diet** | | | | | | | |  |  | | |  |
|  | **CON** | | **BMOS** | | **HMO** | | **BMOS + HMO** | | **Pooled** | ***P*-Value^2^** | | |  |
| **Measure^3^** | **N** | **Mean** | **N** | **Mean** | **N** | **Mean** | **N** | **Mean** | **SEM** | **BMOS** | **HMO** | **INT** | **Accession Number** |
| *5HTR1^4^* | 12 | 0.00^a^ | 12 | -0.49^a^ | 12 | -0.69^a^ | 10 | 0.06^a^ | 0.31 | 0.654 | 0.808 | 0.039 | XM_005672476.2 |
| *5HTR2* | 12 | 0.00 | 12 | -0.04 | 12 | 0.01 | 10 | -0.36 | 0.35 | 0.469 | 0.586 | 0.560 | XM_013980517.1 |
| *5HTR3^5^* | 12 | 0.00 | 12 | -0.91 | 12 | -0.47 | 9 | -0.69 | 0.32 | 0.060 | 0.663 | 0.253 | XM_003357301.3 |
| *5HTR4* | 12 | 0.00 | 12 | 0.01 | 12 | 0.15 | 10 | 0.07 | 0.33 | 0.912 | 0.745 | 0.875 | NM_001001267.1 |
| *5HTR6* | 12 | 0.00 | 12 | -0.42 | 12 | -0.08 | 10 | 0.30 | 0.32 | 0.944 | 0.278 | 0.181 | XM_003356173.3 |
| *5HTR7* | 12 | 0.00 | 12 | -0.04 | 12 | -0.23 | 10 | 0.11 | 0.32 | 0.622 | 0.886 | 0.536 | NM_214085.1 |
| *BDNF* | 12 | 0.00 | 12 | -0.41 | 12 | -0.04 | 10 | -0.39 | 0.32 | 0.219 | 0.965 | 0.922 | XM_005654684.2 |
| *C-FOS* | 12 | 0.00 | 12 | -0.09 | 12 | -0.08 | 10 | -0.05 | 0.39 | 0.905 | 0.946 | 0.825 | NM_001123113.1 |
| *CHRM1* | 12 | 0.00 | 12 | 0.44 | 12 | 0.08 | 10 | 0.14 | 0.39 | 0.265 | 0.634 | 0.404 | NM_214034.1 |
| *CHRM2* | 12 | 0.00 | 12 | 0.24 | 12 | -0.03 | 10 | 0.58 | 0.32 | 0.161 | 0.604 | 0.544 | NM_214261.1 |
| *CHRM3* | 12 | 0.00^ab^ | 11 | -0.59^ab^ | 12 | -0.70^b^ | 10 | 0.39^a^ | 0.29 | 0.366 | 0.612 | 0.004 | NM_001123098.1 |
| *CHRM4^5^* | 12 | 0.00 | 12 | -0.25 | 12 | -0.60 | 10 | 0.21 | 0.32 | 0.339 | 0.807 | 0.072 | XM_003122828.4 |
| *CHRM5* | 12 | 0.00 | 11 | -0.03 | 12 | 0.16 | 9 | -0.38 | 0.34 | 0.363 | 0.751 | 0.410 | XM_013997263.1 |
| *CHRNA2* | 11 | 0.00 | 12 | 0.04 | 12 | -0.08 | 10 | 0.19 | 0.33 | 0.619 | 0.913 | 0.710 | XM_003132824.3 |
| *CHRNA3^5^* | 12 | 0.00 | 12 | 0.41 | 12 | 0.16 | 10 | 0.09 | 0.34 | 0.584 | 0.793 | 0.430 | XM_013988980.1 |
| *CHRNA7* | 12 | 0.00 | 12 | 0.23 | 12 | 0.18 | 10 | 0.31 | 0.33 | 0.559 | 0.671 | 0.863 | XM_013993241.1 |
| *CHRNB2^5^* | 12 | 0.00 | 12 | 0.37 | 12 | 0.46 | 10 | 0.92 | 0.31 | 0.156 | 0.086 | 0.875 | XM_003125722.3 |
| *CHRNB4* | 12 | 0.00 | 12 | 0.19 | 12 | 0.16 | 10 | 0.38 | 0.33 | 0.496 | 0.569 | 0.952 | XM_013988981.1 |
| *CREB* | 12 | 0.00 | 12 | 0.65 | 12 | -0.10 | 10 | 0.07 | 0.32 | 0.155 | 0.241 | 0.407 | NM_001099929.1 |
| *CREBBP* | 12 | 0.00 | 12 | 0.23 | 12 | -0.24 | 10 | 0.53 | 0.31 | 0.098 | 0.905 | 0.362 | XM_003354647.4 |
| *DLG4* | 12 | 0.00 | 11 | 0.37 | 12 | -0.03 | 10 | 0.59 | 0.32 | 0.103 | 0.752 | 0.666 | XR_001303340.1 |
| *DRD1* | 12 | 0.00 | 12 | 0.28 | 12 | -0.19 | 10 | 0.34 | 0.34 | 0.163 | 0.814 | 0.674 | XM_005672535.2 |
| ^1^Abbreviations: CON, control group; BMOS, pigs fed bovine milk oligosaccharides; HMO, pigs fed 2’fucosyllactose and Lacto-N-neotetraose; SEM, standard error of the mean; Int, interaction effect of BMOS and HMO. | | | | | | | | | | | | | |
| ^2^Data analyzed via two-way ANOVA with post-hoc Tukey adjustment for multiple comparisons. | | | | | | | | | | | | | |
| ^3^Standardized values for mRNA expression (mean = 0, standard deviation = 1) centered by control group. | | | | | | | | | | | | | |
| ^4^Mean separation insignificant after Tukey adjustment. | | | | | | | | | | | | | |
| ^5^Number of samples below threshold (median of negative controls): *5HTR3*, 12; *CHRM4*, 6; *CHRNA3*, 5; *CHRNB2*, 4; *DRD4*, 22; *DRD5*, 2; *GLRA4*, 10; *GRIN2C*, 4; *SLC18A2*, 7; *SLC18A3*, 2; *SLC6A2*, 17; *SLC6A3*, 14; *SLC6A4*, 4. | | | | | | | | | | | | | |
| ^abc^Means without a common superscript differ (P < 0.05). | | | | | | | | | | | | | |

| **Supplemental Table 6**. Continued^1^ | | | | | | | | | | | | | |
| --- | --- | --- | --- | --- | --- | --- | --- | --- | --- | --- | --- | --- | --- |
|  | **Diet** | | | | | | | |  |  | | |  |
|  | **CON** | | **BMOS** | | **HMO** | | **BMOS + HMO** | | **Pooled** | ***P*-Value^2^** | | |  |
| **Measure^3^** | **N** | **Mean** | **N** | **Mean** | **N** | **Mean** | **N** | **Mean** | **SEM** | **BMOS** | **HMO** | **INT** | **Accession Number** |
| *DRD2* | 12 | 0.00 | 12 | 0.37 | 12 | -0.24 | 10 | -0.36 | 0.31 | 0.681 | 0.110 | 0.408 | XM_005667325.2 |
| *DRD3* | 12 | 0.00 | 12 | 0.36 | 12 | 0.10 | 10 | 0.62 | 0.32 | 0.150 | 0.549 | 0.795 | XM_013982425.1 |
| *DRD4^5^* | 12 | 0.00 | 12 | -1.00 | 12 | -0.82 | 10 | -0.69 | 0.33 | 0.134 | 0.367 | 0.051 | XM_003122390.2 |
| *DRD5^5^* | 12 | 0.00 | 12 | -0.38 | 12 | -0.56 | 10 | -0.56 | 0.32 | 0.537 | 0.229 | 0.527 | XM_013989284.1 |
| *EGR1* | 12 | 0.00 | 12 | -0.06 | 12 | -0.15 | 10 | 0.23 | 0.37 | 0.573 | 0.816 | 0.444 | XM_003123974.5 |
| *GABBR1* | 12 | 0.00 | 12 | 0.26 | 12 | 0.00 | 10 | 0.52 | 0.32 | 0.198 | 0.678 | 0.667 | NM_001123114.1 |
| *GABBR2* | 12 | 0.00 | 12 | -0.35 | 12 | -0.28 | 10 | -0.09 | 0.32 | 0.787 | 0.984 | 0.374 | XM_003122032.5 |
| *GABRA1* | 12 | 0.00 | 12 | 0.16 | 12 | 0.04 | 9 | 0.94 | 0.30 | 0.069 | 0.155 | 0.198 | XM_013984876.1 |
| *GABRA2* | 12 | 0.00 | 12 | -0.05 | 12 | -0.35 | 10 | -0.65 | 0.32 | 0.557 | 0.119 | 0.672 | XM_013978645.1 |
| *GABRA5* | 12 | 0.00 | 12 | 0.12 | 12 | 0.35 | 10 | -0.27 | 0.32 | 0.415 | 0.948 | 0.228 | XM_005654471.2 |
| *GABRB2* | 12 | 0.00^ab^ | 12 | -0.31^b^ | 12 | -0.30^b^ | 10 | 0.99^a^ | 0.28 | 0.071 | 0.064 | 0.004 | XM_013984878.1 |
| *GABRD* | 12 | 0.00 | 12 | -0.03 | 12 | 0.09 | 9 | 0.88 | 0.32 | 0.164 | 0.067 | 0.131 | XM_013988717.1 |
| *GABRG2* | 12 | 0.00 | 12 | 0.18 | 12 | -0.03 | 9 | 0.35 | 0.32 | 0.358 | 0.827 | 0.750 | XM_003359825.3 |
| *GABRR1*^4^ | 12 | 0.00^a^ | 12 | -0.12^a^ | 12 | -0.70^a^ | 10 | -0.61^a^ | 0.31 | 0.959 | 0.047 | 0.724 | XM_013992686.1 |
| *GAD* | 12 | 0.00 | 12 | 0.04 | 12 | -0.58 | 9 | 0.17 | 0.33 | 0.166 | 0.424 | 0.213 | NM_213894.1 |
| *GLRA1* | 12 | 0.00 | 11 | -0.07 | 12 | -0.28 | 10 | -0.19 | 0.34 | 0.961 | 0.500 | 0.782 | XM_013984909.1 |
| *GLRA2* | 12 | 0.00 | 12 | -0.48 | 12 | -0.59 | 9 | -0.26 | 0.32 | 0.809 | 0.535 | 0.179 | XM_013985912.1 |
| *GLRA3* | 12 | 0.00 | 12 | 0.27 | 12 | 0.55 | 10 | -0.07 | 0.32 | 0.576 | 0.736 | 0.148 | XM_003132832.5 |
| *GLRA4^5^* | 12 | 0.00^ab^ | 12 | -0.45^b^ | 12 | -0.07^ab^ | 10 | 0.70^a^ | 0.32 | 0.548 | 0.048 | 0.027 | XM_003135271.3 |
| *GRIA1* | 12 | 0.00 | 12 | 0.08 | 12 | 0.24 | 10 | -0.32 | 0.32 | 0.433 | 0.785 | 0.292 | XM_003359841.4 |
| *GRIA2* | 12 | 0.00 | 11 | 0.21 | 12 | -0.08 | 10 | -0.04 | 0.33 | 0.693 | 0.589 | 0.768 | XM_005656504.2 |
| *GRIA3* | 12 | 0.00 | 12 | 0.31 | 12 | 0.15 | 10 | 0.70 | 0.32 | 0.156 | 0.370 | 0.686 | XM_003135356.4 |
| *GRIA4* | 12 | 0.00 | 11 | -0.27 | 12 | -0.17 | 10 | 0.55 | 0.36 | 0.414 | 0.242 | 0.080 | XM_013979342.1 |
| ^1^Abbreviations: CON, control group; BMOS, pigs fed bovine milk oligosaccharides; HMO, pigs fed 2’fucosyllactose and Lacto-N-neotetraose; SEM, standard error of the mean; Int, interaction effect of BMOS and HMO. | | | | | | | | | | | | | |
| ^2^Data analyzed via two-way ANOVA with post-hoc Tukey adjustment for multiple comparisons. | | | | | | | | | | | | | |
| ^3^Standardized values for mRNA expression (mean = 0, standard deviation = 1) centered by control group. | | | | | | | | | | | | | |
| ^4^Mean separation insignificant after Tukey adjustment. | | | | | | | | | | | | | |
| ^5^Number of samples below threshold (median of negative controls): *5HTR3*, 12; *CHRM4*, 6; *CHRNA3*, 5; *CHRNB2*, 4; *DRD4*, 22; *DRD5*, 2; *GLRA4*, 10; *GRIN2C*, 4; *SLC18A2*, 7; *SLC18A3*, 2; *SLC6A2*, 17; *SLC6A3*, 14; *SLC6A4*, 4. | | | | | | | | | | | | | |
| ^abc^Means without a common superscript differ (P < 0.05). | | | | | | | | | | | | | |

| **Supplemental Table 6**. Continued ^1^ | | | | | | | | | | | | | |
| --- | --- | --- | --- | --- | --- | --- | --- | --- | --- | --- | --- | --- | --- |
|  | **Diet** | | | | | | | |  |  | | |  |
|  | **CON** | | **BMOS** | | **HMO** | | **BMOS + HMO** | | **Pooled** | ***P*-Value^2^** | | |  |
| **Measure^3^** | **N** | **Mean** | **N** | **Mean** | **N** | **Mean** | **N** | **Mean** | **SEM** | **BMOS** | **HMO** | **INT** | **Accession Number** |
| *GRIN1*^4^ | 11 | 0.00^a^ | 12 | 0.45^a^ | 12 | 0.02^a^ | 10 | 0.53^a^ | 0.37 | 0.035 | 0.818 | 0.888 | XM_013992346.1 |
| *GRIN2A* | 12 | 0.00 | 12 | 0.28 | 11 | 0.15 | 10 | -0.01 | 0.32 | 0.843 | 0.818 | 0.467 | XM_003481048.3 |
| *GRIN2B* | 12 | 0.00 | 12 | -0.04 | 12 | -0.08 | 10 | -0.32 | 0.32 | 0.651 | 0.547 | 0.743 | XM_003355567.3 |
| *GRIN2C^5^* | 12 | 0.00 | 12 | -0.44 | 12 | -0.28 | 10 | 0.21 | 0.33 | 0.928 | 0.529 | 0.113 | XM_013980765.1 |
| *GRIN2D*^4^ | 12 | 0.00^a^ | 12 | -0.62^a^ | 12 | -0.56^a^ | 10 | -0.05^a^ | 0.34 | 0.822 | 0.979 | 0.037 | XM_003127274.4 |
| *HDAC1* | 12 | 0.00 | 12 | 0.00 | 12 | 0.01 | 10 | 0.26 | 0.34 | 0.694 | 0.665 | 0.691 | XM_013999116.1 |
| *HDAC2* | 12 | 0.00 | 12 | 0.27 | 12 | -0.02 | 10 | -0.17 | 0.32 | 0.830 | 0.449 | 0.499 | XM_001925318.5 |
| *HDAC3* | 12 | 0.00 | 12 | -0.40 | 12 | -0.63 | 10 | 0.11 | 0.32 | 0.563 | 0.837 | 0.056 | XM_013995254.1 |
| *HDAC4* | 12 | 0.00 | 12 | -0.18 | 12 | -0.67 | 10 | -0.18 | 0.32 | 0.602 | 0.259 | 0.269 | XM_005657593.2 |
| *HDAC5*^4^ | 12 | 0.00^a^ | 11 | 0.62^a^ | 12 | 0.17^a^ | 10 | 0.87^a^ | 0.35 | 0.015 | 0.428 | 0.876 | XM_013980921.1 |
| *HDAC7* | 12 | 0.00 | 12 | 0.35 | 12 | -0.36 | 10 | 0.20 | 0.35 | 0.117 | 0.379 | 0.707 | XM_013988449.1 |
| *HDAC8* | 12 | 0.00 | 12 | -0.08 | 12 | -0.70 | 10 | -0.22 | 0.32 | 0.491 | 0.156 | 0.335 | XM_013990927.1 |
| *HDAC9* | 12 | 0.00 | 12 | -0.35 | 12 | -0.28 | 10 | 0.23 | 0.36 | 0.776 | 0.603 | 0.142 | XM_013979765.1 |
| *HOMER1* | 12 | 0.00 | 12 | -0.04 | 12 | -0.47 | 10 | -0.18 | 0.32 | 0.675 | 0.318 | 0.575 | NM_001243811.1 |
| IGF1 | 12 | 0.00 | 12 | -0.63 | 12 | -0.68 | 10 | -0.38 | 0.34 | 0.535 | 0.436 | 0.095 | NM_214256.1 |
| IGF2 | 12 | 0.00 | 12 | -0.84 | 12 | -0.98 | 9 | -0.78 | 0.32 | 0.257 | 0.106 | 0.070 | NM_213883.2 |
| *MAG* | 12 | 0.00 | 11 | 0.39 | 12 | 0.45 | 10 | 0.57 | 0.32 | 0.392 | 0.300 | 0.658 | XM_013998313.1 |
| *MBP* | 12 | 0.00 | 12 | 0.39 | 12 | 0.42 | 10 | 0.55 | 0.32 | 0.394 | 0.340 | 0.662 | NM_001001546.2 |
| *NCAM1*^4^ | 12 | 0.00^a^ | 12 | -0.48^a^ | 12 | -0.50^a^ | 10 | 0.21^a^ | 0.33 | 0.682 | 0.743 | 0.041 | XM_005667343.2 |
| *NPY* | 12 | 0.00 | 12 | -0.39 | 12 | -0.09 | 9 | 0.16 | 0.32 | 0.811 | 0.456 | 0.304 | NM_001256367.1 |
| *NR3C1* | 12 | 0.00 | 12 | 0.27 | 12 | 0.27 | 9 | 0.43 | 0.32 | 0.485 | 0.485 | 0.844 | NM_001008481.1 |
| *NR3C2* | 11 | 0.00 | 12 | -0.05 | 12 | 0.16 | 8 | -0.45 | 0.32 | 0.282 | 0.681 | 0.359 | XM_013978843.1 |
| *NR4A1* | 11 | 0.00 | 12 | 0.52 | 12 | 0.26 | 10 | 0.55 | 0.32 | 0.190 | 0.643 | 0.696 | FJ548761.1 |
| ^1^Abbreviations: CON, control group; BMOS, pigs fed bovine milk oligosaccharides; HMO, pigs fed 2’fucosyllactose and Lacto-N-neotetraose; SEM, standard error of the mean; Int, interaction effect of BMOS and HMO. | | | | | | | | | | | | | |
| ^2^Data analyzed via two-way ANOVA with post-hoc Tukey adjustment for multiple comparisons. | | | | | | | | | | | | | |
| ^3^Standardized values for mRNA expression (mean = 0, standard deviation = 1) centered by control group. | | | | | | | | | | | | | |
| ^4^Mean separation insignificant after Tukey adjustment. | | | | | | | | | | | | | |
| ^5^Number of samples below threshold (median of negative controls): *5HTR3*, 12; *CHRM4*, 6; *CHRNA3*, 5; *CHRNB2*, 4; *DRD4*, 22; *DRD5*, 2; *GLRA4*, 10; *GRIN2C*, 4; *SLC18A2*, 7; *SLC18A3*, 2; *SLC6A2*, 17; *SLC6A3*, 14; *SLC6A4*, 4. | | | | | | | | | | | | | |
| ^abc^Means without a common superscript differ (P < 0.05). | | | | | | | | | | | | | |

| **Supplemental Table 6**. Continued ^1^ | | | | | | | | | | | | | |
| --- | --- | --- | --- | --- | --- | --- | --- | --- | --- | --- | --- | --- | --- |
|  | **Diet** | | | | | | | |  |  | | |  |
|  | **CON** | | **BMOS** | | **HMO** | | **BMOS + HMO** | | **Pooled** | ***P*-Value^2^** | | |  |
| **Measure^3^** | **N** | **Mean** | **N** | **Mean** | **N** | **Mean** | **N** | **Mean** | **SEM** | **BMOS** | **HMO** | **INT** | **Accession Number** |
| *NR4A2*^4^ | 12 | 0.00^a^ | 11 | -0.37^a^ | 12 | -0.52^a^ | 10 | 0.56^a^ | 0.30 | 0.211 | 0.480 | 0.015 | NM_001190276.1 |
| *PLP* | 12 | 0.00 | 12 | 0.14 | 12 | 0.25 | 10 | 0.52 | 0.32 | 0.503 | 0.306 | 0.831 | NM_213974.1 |
| *PP1AC* | 12 | 0.00 | 12 | 0.12 | 12 | -0.48 | 10 | 0.14 | 0.35 | 0.166 | 0.389 | 0.341 | NM_001044559.1 |
| *SIRT1* | 12 | 0.00 | 12 | -0.05 | 12 | 0.18 | 10 | 0.41 | 0.34 | 0.761 | 0.280 | 0.631 | NM_001145750.1 |
| *SLC17A6*^4^ | 11 | 0.00^a^ | 12 | -0.58^a^ | 12 | -0.48^a^ | 9 | 0.32^a^ | 0.34 | 0.673 | 0.437 | 0.012 | XM_003122911.4 |
| *SLC17A7* | 12 | 0.00 | 12 | 0.07 | 12 | -0.03 | 10 | -0.11 | 0.40 | 0.977 | 0.620 | 0.713 | XM_003127311.3 |
| *SLC17A8* | 12 | 0.00 | 12 | 0.18 | 12 | 0.19 | 10 | -0.62 | 0.31 | 0.287 | 0.303 | 0.098 | XM_003126690.5 |
| *SLC18A1* | 12 | 0.00 | 12 | -0.22 | 12 | 0.00 | 10 | 0.48 | 0.33 | 0.651 | 0.233 | 0.232 | XM_013990243.1 |
| *SLC18A2*^4,5^ | 12 | 0.00^a^ | 12 | -0.47^a^ | 12 | -0.99^a^ | 10 | -0.76^a^ | 0.30 | 0.670 | 0.031 | 0.230 | NM_001315654.1 |
| *SLC18A3^5^* | 12 | 0.00 | 12 | 0.13 | 12 | 0.28 | 9 | 0.70 | 0.32 | 0.359 | 0.158 | 0.623 | XM_013983446.1 |
| *SLC1A1* | 12 | 0.00 | 12 | 0.48 | 12 | 0.05 | 10 | 0.31 | 0.32 | 0.226 | 0.838 | 0.706 | NM_001164649.1 |
| *SLC1A2* | 12 | 0.00 | 12 | -0.04 | 12 | 0.17 | 9 | 0.87 | 0.31 | 0.247 | 0.064 | 0.198 | XM_003480708.3 |
| *SLC1A3* | 12 | 0.00 | 12 | -0.03 | 12 | -0.26 | 10 | 0.57 | 0.31 | 0.178 | 0.553 | 0.145 | NM_001244425.2 |
| *SLC1A6* | 12 | 0.00 | 12 | 0.57 | 12 | 0.02 | 10 | 0.20 | 0.36 | 0.135 | 0.475 | 0.426 | XM_003123429.4 |
| *SLC1A7* | 11 | 0.00^a^ | 12 | -0.11^a^ | 12 | -0.14^a^ | 10 | -1.23^b^ | 0.32 | 0.026 | 0.020 | 0.066 | XM_013999377.1 |
| *SLC32A1* | 12 | 0.00 | 12 | -0.25 | 12 | -0.25 | 10 | 0.24 | 0.33 | 0.684 | 0.692 | 0.221 | XM_003134437.4 |
| *SLC6A1* | 12 | 0.00 | 12 | -0.45 | 12 | -0.35 | 9 | -0.15 | 0.32 | 0.681 | 0.927 | 0.287 | XM_005669778.2 |
| *SLC6A11* | 12 | 0.00 | 12 | -0.34 | 12 | -0.35 | 10 | 0.34 | 0.31 | 0.549 | 0.576 | 0.088 | XM_005657080.1 |
| *SLC6A13* | 12 | 0.00 | 12 | -0.91 | 12 | -0.98 | 10 | -0.74 | 0.32 | 0.242 | 0.167 | 0.051 | XM_003126576.4 |
| *SLC6A2^5^* | 12 | 0.00 | 11 | -0.55 | 12 | -0.75 | 10 | -0.73 | 0.33 | 0.380 | 0.126 | 0.333 | XM_013994585.1 |
| *SLC6A3*^4,5^ | 12 | 0.00^a^ | 12 | 0.28^a^ | 12 | -0.57^a^ | 10 | -0.62^a^ | 0.34 | 0.689 | 0.018 | 0.593 | XM_005674638.2 |
| *SLC6A4^5^* | 12 | 0.00 | 11 | -0.34 | 12 | 0.36 | 10 | 0.37 | 0.31 | 0.575 | 0.080 | 0.565 | XM_013981192.1 |
| *SNAP25*^4^ | 12 | 0.00^a^ | 12 | -0.58^a^ | 12 | -0.54^a^ | 10 | 0.32^a^ | 0.30 | 0.615 | 0.536 | 0.015 | XM_003359900.3 |
| *SYP* | 12 | 0.00 | 12 | 0.03 | 12 | 0.17 | 10 | 0.18 | 0.34 | 0.953 | 0.596 | 0.968 | XM_003135078.4 |
| *UBE3A* | 12 | 0.00 | 12 | 0.12 | 12 | -0.18 | 9 | -0.33 | 0.33 | 0.960 | 0.295 | 0.645 | NM_001243181.2 |
| ^1^Abbreviations: CON, control group; BMOS, pigs fed bovine milk oligosaccharides; HMO, pigs fed 2’fucosyllactose and Lacto-N-neotetraose; SEM, standard error of the mean; Int, interaction effect of BMOS and HMO. | | | | | | | | | | | | | |
| ^2^Data analyzed via two-way ANOVA with post-hoc Tukey adjustment for multiple comparisons. | | | | | | | | | | | | | |
| ^3^Standardized values for mRNA expression (mean = 0, standard deviation = 1) centered by control group. | | | | | | | | | | | | | |
| ^4^Mean separation insignificant after Tukey adjustment. | | | | | | | | | | | | | |
| ^5^Number of samples below threshold (median of negative controls): *5HTR3*, 12; *CHRM4*, 6; *CHRNA3*, 5; *CHRNB2*, 4; *DRD4*, 22; *DRD5*, 2; *GLRA4*, 10; *GRIN2C*, 4; *SLC18A2*, 7; *SLC18A3*, 2; *SLC6A2*, 17; *SLC6A3*, 14; *SLC6A4*, 4. | | | | | | | | | | | | | |
| ^abc^Means without a common superscript differ (P < 0.05). | | | | | | | | | | | | | |

| **Supplemental Table 6**. Continued^1^ | | | | | | | | | | | | | |
| --- | --- | --- | --- | --- | --- | --- | --- | --- | --- | --- | --- | --- | --- |
|  | **Diet** | | | | | | | |  |  | | |  |
|  | **CON** | | **BMOS** | | **HMO** | | **BMOS + HMO** | | **Pooled** | ***P*-Value^2^** | | |  |
| **Measure^3^** | **N** | **Mean** | **N** | **Mean** | **N** | **Mean** | **N** | **Mean** | **SEM** | **BMOS** | **HMO** | **INT** | **Accession Number** |
| Housekeeping Genes |  |  |  |  |  |  |  |  |  |  |  |  |  |
| *ACTB* | 12 | 0.00 | 12 | 0.57 | 12 | 0.02 | 10 | -0.01 | 0.33 | 0.356 | 0.337 | 0.308 | XM_003124280.4 |
| *GAPDH* | 12 | 0.00 | 12 | -0.17 | 12 | -0.53 | 10 | -0.21 | 0.34 | 0.796 | 0.325 | 0.402 | NM_001206359.1 |
| *GUS* | 12 | 0.00 | 12 | -0.30 | 12 | 0.42 | 10 | -0.65 | 0.30 | 0.020 | 0.899 | 0.181 | NM_001123121.1 |
| *RPL19* | 12 | 0.00 | 12 | 0.45 | 12 | -0.08 | 10 | 0.83 | 0.31 | 0.023 | 0.594 | 0.421 | XM_003131509.4 |
| *RPS18* | 12 | 0.00 | 12 | 0.46 | 12 | 0.47 | 10 | 1.15 | 0.33 | 0.034 | 0.030 | 0.671 | NM_213940.1 |
| *TUBB* | 11 | 0.00 | 12 | -0.02 | 12 | -0.52 | 10 | 0.48 | 0.33 | 0.065 | 0.948 | 0.057 | NM_001044612.1 |
| ^1^Abbreviations: CON, control group; BMOS, pigs fed bovine milk oligosaccharides; HMO, pigs fed 2’fucosyllactose and Lacto-N-neotetraose; SEM, standard error of the mean; Int, interaction effect of BMOS and HMO. | | | | | | | | | | | | | |
| ^2^Data analyzed via two-way ANOVA with post-hoc Tukey adjustment for multiple comparisons. | | | | | | | | | | | | | |
| ^3^Standardized values for mRNA expression (mean = 0, standard deviation = 1) centered by control group. | | | | | | | | | | | | | |
| ^4^Mean separation insignificant after Tukey adjustment. | | | | | | | | | | | | | |
| ^abc^Means without a common superscript differ (P < 0.05). | | | | | | | | | | | | | |

| **Supplemental Table 7**. Correlations between the recognition index after a 1-h delay and significant MRI and gene expression outcomes^1,2^ | | | | | | | | | | |
| --- | --- | --- | --- | --- | --- | --- | --- | --- | --- | --- |
|  | **CON** | | **BMOS** | | **HMO** | | **BMOS + HMO** | | **Overall** | |
| **Measure** | ***r*** | ***P*** | ***r*** | ***P*** | ***r*** | ***P*** | ***r*** | ***P*** | ***β_1_*** | ***P*** |
| 1-h Delay |  |  |  |  |  |  |  |  |  |  |
| *5HTR1* | -0.22 | 0.58 | 0.10 | 0.79 | 0.01 | 0.98 | 0.10 | 0.78 | 0.00 | 0.90 |
| *GABRB2* | -0.32 | 0.40 | -0.12 | 0.74 | 0.05 | 0.89 | 0.11 | 0.76 | 0.00 | 0.99 |
| *GABRR1* | -0.27 | 0.48 | -0.44 | 0.21 | 0.12 | 0.73 | -0.60 | 0.07 | -0.08 | 0.01 |
| *GRIN1* | -0.89 | 0.00 | -0.13 | 0.72 | -0.52 | 0.12 | -0.34 | 0.34 | -0.08 | 0.01 |
| *GRIN2D* | -0.47 | 0.20 | 0.37 | 0.29 | -0.38 | 0.27 | -0.07 | 0.84 | -0.02 | 0.63 |
| *HDAC5* | -0.78 | 0.01 | 0.18 | 0.62 | -0.54 | 0.11 | -0.68 | 0.04 | -0.07 | 0.03 |
| *NCAM1* | 0.12 | 0.76 | 0.58 | 0.08 | -0.29 | 0.41 | 0.16 | 0.66 | 0.04 | 0.24 |
| *NR4A2* | -0.32 | 0.40 | 0.16 | 0.65 | -0.31 | 0.41 | 0.06 | 0.87 | -0.01 | 0.75 |
| *SLC17A6* | 0.10 | 0.80 | 0.36 | 0.31 | -0.17 | 0.64 | 0.14 | 0.70 | 0.02 | 0.54 |
| *SLC18A2* | 0.23 | 0.55 | 0.32 | 0.40 | -0.08 | 0.83 | 0.37 | 0.30 | 0.02 | 0.51 |
| *SLC1A7* | -0.57 | 0.11 | -0.13 | 0.73 | -0.39 | 0.27 | -0.37 | 0.33 | -0.07 | 0.03 |
| *SLC6A3* | -0.51 | 0.20 | 0.36 | 0.31 | 0.05 | 0.89 | 0.25 | 0.56 | -0.02 | 0.47 |
| *SNAP25* | -0.09 | 0.81 | 0.40 | 0.25 | -0.27 | 0.45 | -0.05 | 0.89 | 0.00 | 0.94 |
| *CHRM3* | 0.03 | 0.94 | 0.17 | 0.64 | 0.06 | 0.87 | 0.38 | 0.28 | 0.03 | 0.44 |
| *GLRA4* | -0.44 | 0.24 | 0.11 | 0.76 | -0.36 | 0.30 | 0.44 | 0.20 | 0.02 | 0.54 |
| *RPL19* | -0.11 | 0.77 | 0.15 | 0.68 | 0.32 | 0.37 | 0.16 | 0.66 | 0.03 | 0.43 |
| *RPS18* | -0.04 | 0.91 | 0.05 | 0.89 | 0.31 | 0.38 | -0.04 | 0.91 | 0.02 | 0.49 |
| Grey Matter, mm^3^ | 0.41 | 0.32 | -0.02 | 0.96 | 0.45 | 0.26 | 0.35 | 0.35 | 0.00 | 0.40 |
| Hypothalamus, mm^3^ | 0.65 | 0.08 | -0.46 | 0.22 | 0.61 | 0.14 | -0.09 | 0.83 | 0.00 | 0.99 |
| ^1^Abbreviations: mm, millimeter; s, second; CON, control group; BMOS, pigs fed bovine milk oligosaccharides; HMO, pigs fed 2’fucosyllactose and Lacto-N-neotetraose; Int, interaction effect of BMOS and HMO; *r;* Pearson correlation coefficient; *P*, P-value; *β*1, slope of linear regression equation; mm, millimeter; %TBV, percent total brain volume. | | | | | | | | | | |
| ^2^Correlations were performed using only the outcomes significantly affected by diet for MRI and gene expression outcomes against the recognition index after a 1- or 48-h delay. Correlations are presented by diet group, whereas diet was not included in the linear regression model to estimate the diet independent relationship between outcomes. P-values indicate whether the Pearson correlation coefficient or slope are significantly different from zero, indicating a linear relationship between outcomes. | | | | | | | | | | |

| **Supplemental Table 7**. Continued^1,2^ | | | | | | | | | | |
| --- | --- | --- | --- | --- | --- | --- | --- | --- | --- | --- |
|  | **CON** | | **BMOS** | | **HMO** | | **BMOS + HMO** | | **Overall** | |
| **Measure** | ***r*** | ***P*** | ***r*** | ***P*** | ***r*** | ***P*** | ***r*** | ***P*** | ***β_1_*** | ***P*** |
| 1-h Delay |  |  |  |  |  |  |  |  |  |  |
| Lateral Ventricle, mm^3^ | 0.66 | 0.08 | -0.11 | 0.76 | 0.20 | 0.63 | 0.23 | 0.55 | 0.00 | 0.66 |
| Thalamus, mm^3^ | -0.81 | 0.01 | 0.37 | 0.29 | -0.01 | 0.98 | -0.12 | 0.77 | 0.00 | 0.44 |
| Caudate, %TBV | -0.42 | 0.30 | -0.03 | 0.93 | -0.29 | 0.48 | -0.18 | 0.64 | -1.04 | 0.20 |
| Corpus Callosum, %TBV | 0.64 | 0.09 | 0.24 | 0.54 | 0.28 | 0.50 | -0.19 | 0.62 | 1.46 | 0.12 |
| Lateral Ventricle, %TBV | -0.93 | 0.00 | 0.37 | 0.30 | -0.05 | 0.92 | -0.36 | 0.38 | -0.25 | 0.59 |
| Left Cortex, %TBV | -0.51 | 0.20 | 0.25 | 0.48 | 0.73 | 0.06 | -0.12 | 0.75 | 0.06 | 0.28 |
| Pons, %TBV | 0.26 | 0.54 | -0.28 | 0.44 | 0.03 | 0.95 | 0.69 | 0.04 | 0.31 | 0.48 |
| Right Cortex, %TBV | -0.42 | 0.30 | 0.22 | 0.54 | 0.66 | 0.11 | -0.30 | 0.44 | 0.04 | 0.44 |
| ^1^Abbreviations: mm, millimeter; s, second; CON, control group; BMOS, pigs fed bovine milk oligosaccharides; HMO, pigs fed 2’fucosyllactose and Lacto-N-neotetraose; Int, interaction effect of BMOS and HMO; *r;* Pearson correlation coefficient; *P*, P-value; *β*1, slope of linear regression equation; mm, millimeter; %TBV, percent total brain volume. | | | | | | | | | | |
| ^2^Correlations were performed using only the outcomes significantly affected by diet for MRI and gene expression outcomes against the recognition index after a 1- or 48-h delay. Correlations are presented by diet group, whereas diet was not included in the linear regression model to estimate the diet independent relationship between outcomes. P-values indicate whether the Pearson correlation coefficient or slope are significantly different from zero, indicating a linear relationship between outcomes. | | | | | | | | | | |

| **Supplemental Table 8**. Correlations between the recognition index after a 48-h delay and significant MRI and gene expression outcomes^1,2^ | | | | | | | | | | |
| --- | --- | --- | --- | --- | --- | --- | --- | --- | --- | --- |
|  | **CON** | | **BMOS** | | **HMO** | | **BMOS + HMO** | | **Overall** | |
| **Measure** | ***r*** | ***P*** | ***r*** | ***P*** | ***r*** | ***P*** | ***r*** | ***P*** | ***β_1_*** | ***P*** |
| 48-h Delay |  |  |  |  |  |  |  |  |  |  |
| *5HTR1* | -0.21 | 0.56 | -0.09 | 0.81 | 0.19 | 0.57 | -0.68 | 0.04 | -0.02 | 0.50 |
| *GABRB2* | -0.23 | 0.52 | -0.26 | 0.50 | 0.26 | 0.45 | -0.64 | 0.06 | -0.02 | 0.57 |
| *GABRR1* | -0.06 | 0.88 | 0.20 | 0.60 | -0.01 | 0.98 | 0.29 | 0.45 | 0.02 | 0.56 |
| *GRIN1* | -0.29 | 0.41 | -0.49 | 0.19 | 0.58 | 0.06 | 0.17 | 0.66 | 0.01 | 0.85 |
| *GRIN2D* | 0.07 | 0.86 | -0.04 | 0.93 | 0.17 | 0.62 | -0.33 | 0.38 | 0.00 | 0.99 |
| *HDAC5* | -0.55 | 0.10 | -0.58 | 0.10 | 0.40 | 0.23 | -0.30 | 0.47 | -0.01 | 0.83 |
| *NCAM1* | 0.15 | 0.68 | -0.57 | 0.11 | -0.07 | 0.84 | -0.65 | 0.06 | -0.05 | 0.11 |
| *NR4A2* | 0.22 | 0.53 | -0.79 | 0.01 | 0.20 | 0.58 | -0.79 | 0.01 | -0.03 | 0.34 |
| *SLC17A6* | 0.09 | 0.80 | -0.10 | 0.79 | 0.12 | 0.72 | -0.82 | 0.01 | -0.03 | 0.45 |
| *SLC18A2* | -0.05 | 0.88 | 0.57 | 0.14 | -0.02 | 0.96 | -0.14 | 0.71 | 0.02 | 0.59 |
| *SLC1A7* | 0.20 | 0.59 | -0.66 | 0.05 | 0.26 | 0.44 | 0.62 | 0.10 | -0.01 | 0.85 |
| *SLC6A3* | 0.33 | 0.38 | -0.24 | 0.53 | -0.42 | 0.19 | 0.42 | 0.35 | -0.02 | 0.66 |
| *SNAP25* | 0.03 | 0.94 | -0.12 | 0.76 | 0.04 | 0.90 | -0.01 | 0.98 | 0.01 | 0.87 |
| *CHRM3* | 0.09 | 0.80 | 0.60 | 0.09 | 0.08 | 0.82 | -0.43 | 0.25 | 0.02 | 0.53 |
| *GLRA4* | 0.23 | 0.52 | 0.13 | 0.74 | 0.14 | 0.67 | -0.54 | 0.14 | 0.00 | 0.92 |
| *RPL19* | 0.62 | 0.06 | -0.51 | 0.16 | -0.20 | 0.57 | 0.18 | 0.64 | -0.01 | 0.88 |
| *RPS18* | -0.03 | 0.94 | -0.09 | 0.82 | -0.29 | 0.39 | -0.09 | 0.83 | -0.02 | 0.64 |
| Grey Matter, mm^3^ | 0.07 | 0.87 | 0.00 | 1.00 | -0.10 | 0.79 | 0.69 | 0.06 | 0.00 | 0.61 |
| Hypothalamus, mm^3^ | 0.11 | 0.77 | 0.18 | 0.65 | 0.12 | 0.78 | -0.01 | 0.98 | 0.00 | 0.51 |
| ^1^Abbreviations: mm, millimeter; s, second; CON, control group; BMOS, pigs fed bovine milk oligosaccharides; HMO, pigs fed 2’fucosyllactose and Lacto-N-neotetraose; Int, interaction effect of BMOS and HMO; *r;* Pearson correlation coefficient; *P*, P-value; *β*1, slope of linear regression equation; mm, millimeter; %TBV, percent total brain volume. | | | | | | | | | | |
| ^2^Correlations were performed using only the outcomes significantly affected by diet for MRI and gene expression outcomes against the recognition index after a 1- or 48-h delay. Correlations are presented by diet group, whereas diet was not included in the linear regression model to estimate the diet independent relationship between outcomes. P-values indicate whether the Pearson correlation coefficient or slope are significantly different from zero, indicating a linear relationship between outcomes. | | | | | | | | | | |

| **Supplemental Table 8**. Continued^1,2^ | | | | | | | | | | |
| --- | --- | --- | --- | --- | --- | --- | --- | --- | --- | --- |
|  | **CON** | | **BMOS** | | **HMO** | | **BMOS + HMO** | | **Overall** | |
| **Measure** | ***r*** | ***P*** | ***r*** | ***P*** | ***r*** | ***P*** | ***r*** | ***P*** | ***β_1_*** | ***P*** |
| 48-h Delay |  |  |  |  |  |  |  |  |  |  |
| Lateral Ventricle, mm^3^ | 0.66 | 0.08 | -0.11 | 0.76 | 0.20 | 0.63 | 0.23 | 0.55 | 0.00 | 0.66 |
| Thalamus, mm^3^ | -0.81 | 0.01 | 0.37 | 0.29 | -0.01 | 0.98 | -0.12 | 0.77 | 0.00 | 0.44 |
| Caudate, %TBV | -0.42 | 0.30 | -0.03 | 0.93 | -0.29 | 0.48 | -0.18 | 0.64 | -1.04 | 0.20 |
| Corpus Callosum, %TBV | 0.64 | 0.09 | 0.24 | 0.54 | 0.28 | 0.50 | -0.19 | 0.62 | 1.46 | 0.12 |
| Lateral Ventricle, %TBV | -0.93 | 0.00 | 0.37 | 0.30 | -0.05 | 0.92 | -0.36 | 0.38 | -0.25 | 0.59 |
| Left Cortex, %TBV | -0.51 | 0.20 | 0.25 | 0.48 | 0.73 | 0.06 | -0.12 | 0.75 | 0.06 | 0.28 |
| Pons, %TBV | 0.26 | 0.54 | -0.28 | 0.44 | 0.03 | 0.95 | 0.69 | 0.04 | 0.31 | 0.48 |
| Right Cortex, %TBV | -0.42 | 0.30 | 0.22 | 0.54 | 0.66 | 0.11 | -0.30 | 0.44 | 0.04 | 0.44 |
| ^1^Abbreviations: mm, millimeter; s, second; CON, control group; BMOS, pigs fed bovine milk oligosaccharides; HMO, pigs fed 2’fucosyllactose and Lacto-N-neotetraose; Int, interaction effect of BMOS and HMO; *r;* Pearson correlation coefficient; *P*, P-value; *β*1, slope of linear regression equation; mm, millimeter; %TBV, percent total brain volume. | | | | | | | | | | |
| ^2^Correlations were performed using only the outcomes significantly affected by diet for MRI and gene expression outcomes against the recognition index after a 1- or 48-h delay. Correlations are presented by diet group, whereas diet was not included in the linear regression model to estimate the diet independent relationship between outcomes. P-values indicate whether the Pearson correlation coefficient or slope are significantly different from zero, indicating a linear relationship between outcomes. | | | | | | | | | | |
